# GCN-Transformer: Graph Convolutional Network and Transformer for Multi-Person Pose Forecasting Using Sensor-Based Motion Data

**DOI:** 10.3390/s25103136

**Published:** 2025-05-15

**Authors:** Romeo Šajina, Goran Oreški, Marina Ivašić-Kos

**Affiliations:** 1 Faculty of Informatics and Digital Technologies, University of Rijeka, 51000 Rijeka, Croatia; rsajina@unipu.hr; 2 Faculty of Informatics, Juraj Dobrila University of Pula, 52100 Pula, Croatia; goreski@unipu.hr; 3 Centre for Artificial Intelligence, University of Rijeka, 51000 Rijeka, Croatia

**Keywords:** multi-person pose forecasting, transformer architecture, GCN, GCN-Transformer, SoMoF Benchmark, ExPI dataset

## Abstract

**Highlights:**

This paper presents the GCN-Transformer, a novel deep learning model that integrates Graph Convolutional Networks (GCNs) and Transformers to enhance multi-person pose forecasting. The model effectively captures both spatial and temporal dependencies, improving the performance of pose forecasting. Additionally, a new evaluation metric, Final Joint Position and Trajectory Error (FJPTE), is introduced to provide a more comprehensive assessment of movement dynamics. These contributions establish GCN-Transformer as a state-of-the-art solution in pose forecasting.

**What are the main findings?**
  - We introduce GCN-Transformer, a novel architecture combining Graph Convolutional Networks (GCNs) and Transformers for multi-person pose forecasting.
  - We propose a new evaluation metric, Final Joint Position and Trajectory Error (FJPTE), which comprehensively assesses both local and global movement dynamics.
**What is the implication of the main finding?**
  - GCN-Transformer achieves state-of-the-art performances on the CMU-Mocap, MuPoTS- 3D, SoMoF Benchmark, and ExPI datasets, demonstrating superior generalization across different motion scenarios.
  - The proposed FJPTE metric improves the evaluation of pose forecasting models by accounting for both movement trajectory and final position, enabling better assessments of motion realism.

**Abstract:**

Multi-person pose forecasting involves predicting the future body poses of multiple individuals over time, involving complex movement dynamics and interaction dependencies. Its relevance spans various fields, including computer vision, robotics, human–computer interaction, and surveillance. This task is particularly important in sensor-driven applications, where motion capture systems, including vision-based sensors and IMUs, provide crucial data for analyzing human movement. This paper introduces GCN-Transformer, a novel model for multi-person pose forecasting that leverages the integration of Graph Convolutional Network and Transformer architectures. We integrated novel loss terms during the training phase to enable the model to learn both interaction dependencies and the trajectories of multiple joints simultaneously. Additionally, we propose a novel pose forecasting evaluation metric called Final Joint Position and Trajectory Error (FJPTE), which assesses both local movement dynamics and global movement errors by considering the final position and the trajectory leading up to it, providing a more comprehensive assessment of movement dynamics. Our model uniquely integrates scene-level graph-based encoding and personalized attention-based decoding, introducing a novel architecture for multi-person pose forecasting that achieves state-of-the-art results across four datasets. The model is trained and evaluated on the CMU-Mocap, MuPoTS-3D, SoMoF Benchmark, and ExPI datasets, which are collected using sensor-based motion capture systems, ensuring its applicability in real-world scenarios. Comprehensive evaluations on the CMU-Mocap, MuPoTS-3D, SoMoF Benchmark, and ExPI datasets demonstrate that the proposed GCN-Transformer model consistently outperforms existing state-of-the-art (SOTA) models according to the VIM and MPJPE metrics. Specifically, based on the MPJPE metric, GCN-Transformer shows a 4.7% improvement over the closest SOTA model on CMU-Mocap, 4.3% improvement over the closest SOTA model on MuPoTS-3D, 5% improvement over the closest SOTA model on the SoMoF Benchmark, and a 2.6% improvement over the closest SOTA model on the ExPI dataset. Unlike other models with performances that fluctuate across datasets, GCN-Transformer performs consistently, proving its robustness in multi-person pose forecasting and providing an excellent foundation for the application of GCN-Transformer in different domains.

## 1. Introduction

Pose forecasting is a machine learning task that predicts future poses based on a historical sequence of poses. This task is inherently challenging, as it requires models to anticipate movements several seconds into the future, thereby necessitating the capture of intricate temporal dynamics. The goal of pose forecasting is to provide accurate predictions of future poses, which can have practical applications in a wide range of fields. For example, in robotics, pose forecasting models enable robots to infer human intentions and predict future movements, facilitating safer, more intuitive collaboration in environments such as manufacturing floors, healthcare, and assistive robotics [1,2,3,4,5,6]. In sports analytics, forecasting player trajectories and body orientations several moments ahead supports tactical decision-making, performance evaluation, and even automated highlight generation. In autonomous driving, the accurate prediction of pedestrian motion improves vehicle navigation and enhances safety in complex urban settings. Intelligent surveillance systems use pose forecasting to proactively detect abnormal group behaviors, such as crowd surges or physical altercations, by identifying deviations from expected motion patterns. In virtual and augmented reality, forecasting full-body motion enables latency compensation and smoother avatar rendering during real-time collaborative experiences or immersive gameplay. These applications often rely on sensor-based motion capture systems, including vision-based sensors, inertial measurement units (IMUs), and depth cameras, to collect high-precision human movement data for training and inference [7,8,9].

One way to conceptualize pose forecasting is to divide it into two main categories: single-person [3,10,11,12,13,14] and multi-person [15,16,17,18,19,20] pose forecasting. In single-person pose forecasting, the task focuses on predicting the future poses of an individual based solely on their previous poses. This scenario is typically less complex, as it involves modeling the movement patterns of a single entity. On the other hand, multi-person pose forecasting extends the task by simultaneously predicting the future poses of multiple individuals. In this scenario, the forecasting model needs to consider each person’s previous poses and extract social dependencies and interactions among them. These interactions could include factors such as proximity, response to a movement, and body language, which significantly influence the future movements of individuals within a scene.

Various deep learning methods have been employed to tackle the task of pose forecasting. Fully connected networks directly map input pose sequences to future predictions, which is suitable for straightforward temporal dependencies [10,11,17]. Recurrent neural networks (RNNs) capture long-range dependencies by maintaining hidden states across time steps [12]. Graph Convolutional Networks (GCNs) excel in modeling spatial dependencies and interactions in multi-person scenarios [3,13,20,21]. Attention mechanisms and Transformer architectures focus on the relevant parts of input sequences, handling long-range dependencies effectively for precise predictions [15,16,18,19].

The paper presents a novel model, GCN-Transformer, designed to address the challenges of multi-person pose forecasting. Our model integrates key features from various deep learning architectures to capture complex spatiotemporal dependencies and social interactions among multiple individuals in a scene. GCN-Transformer consists of two main modules: the Scene Module and the Spatiotemporal Attention Forecasting Module. The Scene Module leverages Graph Convolutional Networks (GCNs) to extract social features and dependencies from the scene context, while the Spatiotemporal Attention Forecasting Module utilizes a combination of Temporal Graph Convolutional Networks (T-GCNs) and Transformer decoder modules to predict future poses. By combining these components, GCN-Transformer achieves state-of-the-art performance in multi-person pose forecasting tasks, demonstrating its effectiveness in capturing intricate motion dynamics and social interactions. GCN-Transformer is trained and evaluated on sensor-based datasets CMU-Mocap, MuPoTS-3D, SoMoF Benchmark, and ExPI, which include motion capture data collected through real-world sensing systems. To enhance the learning process and improve the movement dynamics of predicted sequences while also capturing interaction dependencies, we introduce new loss terms during the training phase, specifically the multi-person joint distance loss and velocity loss. These loss terms are designed to encourage the model to learn both interaction dependencies and joint movement dynamics. The inter-individual joint distance loss focuses on maintaining realistic spatial relationships between joints, while velocity loss promotes the accurate modeling of movement dynamics.

Additionally, in this paper, we introduce a novel evaluation metric, Final Joint Position and Trajectory Error (FJPTE), designed to comprehensively assess pose forecasting performance. While several attempts have been made to develop evaluation metrics specifically for pose forecasting [17,19,22], these have predominantly been variations of well-known metrics such as MPJPE and VIM, both of which originate from the pose estimation domain. However, pose forecasting requires a more holistic approach that considers not only the final position of each joint but also the trajectory leading to that position. FJPTE addresses this need by evaluating both the final position and the movement dynamics throughout the trajectory, providing a more thorough assessment of how well a model captures the complexities of human motion over time.

Our contributions are as follows:

- We propose a new architecture and model that combines Graph Convolutional Networks (GCNs) and Transformer modules for multi-person pose forecasting; it is designed to handle complex interactions in dynamic scenes and consistently outperforms state-of-the-art models on standard evaluation metrics.
- Multi-person joint distance loss (MPJD) and Velocity Loss (VL) were designed to encourage the model to generate spatially interaction-dependent and temporally coherent pose sequences for dynamic and interaction-rich scenes.
- A new evaluation metric for pose forecasting, called FJPTE, that evaluates movement trajectories and the final position error, is proposed to better assess the realism and coherency of predicted pose sequences in dynamic and interaction-rich scenes.

In this work, we aim to address the challenge of forecasting future 3D poses in dynamic multi-person scenarios by designing a model that combines scene-level social context encoding with individual-specific forecasting using query token fusion. The architecture jointly models spatial dependencies within each individual and temporal motion patterns using both Transformer and GCN-based components. We evaluate the model across four datasets, CMU Mocap, MuPoTS 3D, SoMoF, and ExPI, which feature varying numbers of individuals and different levels of interaction complexity. This setup allows us to assess the robustness and generalization ability of the model across diverse motion conditions.

The organization of this paper is structured to comprehensively address the advancements and methodologies in multi-person pose forecasting. We begin with a review of the related work by discussing existing models and their limitations. Next, we define the problem formulation for multi-person forecasting, detailing the task’s objectives and the necessary input and output representations. Following this, we introduce our proposed model, GCN-Transformer, which is elaborated through several subsections: the Spatiotemporal Fully Connected module for projecting sequences into a higher-dimensional embedding space; the Scene Module for capturing social interactions; and the Spatiotemporal Attention Forecasting Module for predicting future poses, data preprocessing, and augmentation techniques to enhance model performance, along with the training procedures employed. The Experimental Results Section follows, where we describe the metrics used for evaluation, the datasets involved, and the model’s performance on the CMU-Mocap, MuPoTS-3D, SoMoF Benchmark, and ExPI datasets. We then present an ablation study to analyze the impact of different model components. Additionally, we introduce a novel evaluation metric, FJPTE, which assesses both local movement dynamics and global movement errors. Finally, we conclude the paper by summarizing the key findings and discussing future research directions.

## 2. Related Work

In the domain of pose forecasting, establishing a baseline is crucial, with the Zero-Velocity model serving as a simple yet effective benchmark. This model predicts future poses by duplicating the last observed pose. Remarkably, this baseline has emerged as a strong contender, outperforming numerous proposed models and thus providing a fundamental comparison point. Consequently, this paper exclusively discusses models that surpass this baseline performance.

### 2.1. Single-Person Pose Forecasting

Early explorations [3,10,11,12,13,23,24] focused predominantly on single-person pose forecasting. However, when applied to multi-person scenarios, these models independently conduct pose forecasting for each individual.

The LTD model introduced by Mao et al. in [3] uses a Graph Convolutional Network (GCN) with 12 blocks and residual connections, along with two additional graph convolutional layers placed at the beginning and end of the model to encode temporal information and decode features for pose prediction. The Future Motion model was proposed in [13] for single-person pose forecasting on a similar backbone architecture of 12 GCN blocks and also includes data augmentation, curriculum learning, and the use of Online Hard Keypoints Mining (OHKM) loss.

Parsaeifard et al. in [12] proposed a DViTA model that uses a Long Short-Term Memory (LSTM) encoder–decoder network for trajectory forecasting and a Variational LSTM AutoEncoder (VAE) for local pose dynamic forecasting in order to extract two distinct components of human movement: global trajectory and local pose dynamics.

MotionMixer, introduced by Bouazizi et al. in [11], proposes multi-layer perceptrons (MLPs) for pose forecasting and captures spatiotemporal dependencies through spatial mixing across body joints and temporal mixing across time steps by incorporating squeeze-and-excitation (SE) blocks to adjust the significance of different time steps. Guo et al. in [10] proposed siMLPe, a lightweight MLP-based model for pose forecasting that, in addition to having fully connected layers and carrying out layer normalization and transpose operations, contains a Discrete Cosine Transform (DCT) to encode temporal information and carry out residual displacement to predict motion.

Incorporating additional constraints into the problem’s formulation, such as modeling human–scene interactions using per-joint contact maps to capture the distance between human joints and scene points, can enhance pose forecasting performance, as demonstrated by Mao et al. in [23]. This approach resolves issues such as “ghost motion”, conditioning future human poses on predicted contact points.

Zhong et al. in [24] introduced a model called GAGCN that addresses the complex spatiotemporal dependencies in human motion data. The authors use a gating network to dynamically blend multiple adaptive adjacency matrices that capture joint dependencies (spatial) and temporal correlations.

### 2.2. Multi-Person Pose Forecasting

Recent advancements in multi-person pose forecasting have emphasized the integration of social interactions and dependencies among individuals within a scene, aiming to enhance model performance [15,16,17,18,19,20,25,26,27].

Wang et al. in [15] proposed a transformer-based architecture called the Multi-Range Transformer (MRT) that captures both local individual motion and global social interactions among multiple individuals. The MRT decoder predicts future poses for each person by attending to both local- and global-range encoder features. Additionally, a motion discriminator is incorporated into the training process to ensure the generated motions maintain natural characteristics.

The Transformer Encoder was used in the SoMoFormer model, introduced by Vendrow et al. in [16], which treats each input as a Discrete Cosine Transform (DCT)-encoded, padded trajectory of one joint. The SoMoFormer model simultaneously predicts pose trajectories for multiple individuals and uses attention mechanisms to model human body dynamics and the grid position of individuals for its spatial understanding.

In [17], Šajina and Ivasic-Kos proposed the MPFSIR model, which focuses on spatial and temporal pose information using fully connected layers with skip connections. Despite its relatively low model parameters, MPFSIR achieves state-of-the-art performances. Moreover, the model includes an auxiliary output to recognize social interactions between individuals, contributing to its overall performance improvement.

Xu et al. uses temporal differentiation of joints and explicit joint relations as inputs to a joint-relation transformer model called JRTransformer, introduced in [18], which models future relations between joints along with future joint positions.

TBIFormer, proposed by Peng et al. in [19], breaks down human poses into five body parts and models their interactions separately. It employs a Temporal Body Partition Module to transform sequences into a Multi-Person Body-Part sequence, retaining spatial and temporal information. The subsequent module, Social Body Interaction Self-Attention, aims to learn body part dynamics for both inter-individual and intra-individual interactions. Finally, a Transformer Decoder forecasts future movements based on the extracted features and Global Body Query Tokens.

In [20], Peng et al. proposed SocialTGCN, a convolution-based model comprising a Pose Refine Module (PSM) consisting of Graph Convolutional Network (GCN) layers, a Social Temporal GCN (SocialTGCN) encoder with GCN and Temporal Convolutional Network (TCN) layers, and a TCN decoder. Additionally, the SocialTGCN Module is fed a Spatial Adjacency Matrix constructed based on the Euclidean distance between the body root trajectories of individuals.

In recent years, several innovative approaches have emerged for creating multi-person forecasting models that diverge significantly from traditional approaches, offering new ways to handle the complexities of social interactions and motion dynamics. In the following, we discuss a few notable examples of these alternative approaches.

Jeong et al. in [25] have integrated pose forecasting with trajectory forecasting in their Trajectory2Pose model. This interaction-aware, trajectory-conditioned model first predicts multi-modal global trajectories and then refines local pose predictions based on these trajectories. It utilizes a graph-based person-wise interaction module to model inter-person dynamics and reciprocal forecasting of both global trajectories and local poses for improved prediction performance in multi-person scenarios.

In [26], Tanke et al. proposed a framework for predicting the poses of multiple individuals with mutual interactions that bases the prediction of future movements on past behaviors, and they also proposed a function that aggregates movement features across individuals, either by averaging or using multi-head attention to provide contextually plausible interactions for groups of different sizes. By leveraging causal temporal convolutional networks, the model processes the relationships between participants and generates realistic, socially consistent motions over extended time horizons.

Xu et al. in [27] proposed a framework (DuMMF) for stochastic multi-person pose forecasting that incorporates generative modeling and latent codes to model individual movements at the local level and social interactions at the global level. The model generates multiple different predictions for individual poses and social interactions, covering a range of possible outcomes. The approach is generalizable to various generative models, including GANs and diffusion models.

A prevalent technique in data preprocessing for pose forecasting involves the application of the Discrete Cosine Transform (DCT), which encodes human motion into the frequency domain represented by a set of coefficients. This transformation aids in noise reduction, thus improving the robustness of the data. Conversely, the Inverse DCT (IDCT) decodes predictions back to Cartesian coordinates, facilitating interpretation and application [3,10,13,15,16,19,20,23,25].

To further enhance the performance of pose forecasting models, a strategy often employed is dividing the task into short-term and long-term prediction models, also known as short-term and long-term optimization. In this approach, the final prediction is derived from a combination of outputs from both short-term and long-term models [13,16,18]. Additionally, another effective technique to improve transformer-based models is deep supervision. Here, the output of each block within the model is passed through the decoder model, thereby mitigating issues related to overfitting and enhancing model generalization [16,18].

Despite the advancements in pose forecasting, including substantial advancements driven by GCN and Transformer architectures, several limitations persist that challenge the field. Current models often produce structurally invalid poses, where predicted poses do not reflect anatomically feasible configurations, rendering them unrealistic or impossible in real-world settings. Additionally, many models struggle to capture natural movement dynamics, leading to “ghosting” effects where poses appear frozen or drift unrealistically and lacking the fluidity and continuity expected in human motion. A further important issue is generalizability, where certain models achieve strong performance on specific datasets but frequently underperform when tested on different datasets, indicating an over-reliance on dataset-specific characteristics. To address these challenges, our proposed model is designed to improve the structural validity of predicted poses, enhance the realism of movement dynamics, and achieve more consistent performance across diverse datasets.

### 2.3. Pose Forecasting Evaluation Metrics

The evaluation of pose forecasting models involves adopting various metrics borrowed from related tasks, such as pose estimation [28,29]. Initially, the Mean Per Joint Position Error (MPJPE) metric, borrowed from pose estimation, was widely used. However, it calculates the Euclidean distance (L2 norm) across all joints in the predicted sequence, providing an overall assessment of the model’s performance without specifically focusing on human movement dynamics. To address this limitation, Adeli et al. in [22] introduced the Visibility-Ignored Metric (VIM). Unlike MPJPE, VIM evaluates the pose error solely at the last predicted frame, overlooking the trajectory of joints in preceding frames and focusing solely on the final pose error. MPJPE, along with VIM, has since become a standard evaluation metric for pose forecasting due to its simplicity, interpretability, and broad adoption in recent works.

Building upon the MPJPE metric, Šajina and Ivasic-Kos in [17] proposed the Movement-Weighted Mean Per Joint Position Error (MW-MPJPE). This metric enhances MPJPE by incorporating a weighting factor based on the overall movement exhibited by the individual throughout the target pose sequence. This weighting factor provides a more nuanced evaluation by considering the varying degrees of movement across different poses.

Peng et al. in [19] employed various evaluation metrics to assess multi-person pose forecasting models. These included the Joint Position Error (JPE), which resembles MPJPE but reports errors for all individuals in the scene; the Aligned Mean Per Joint Position Error (APE), which is akin to Root-MPJPE, focusing on pose position errors by removing global movement; and the Final Displacement Error (FDE), measuring the trajectory prediction error by considering only the final global position (e.g., pelvis) of each person.

Despite the introduction of several evaluation metrics, most existing metrics either focus solely on joint-wise positional errors or isolate specific aspects of motion, such as the final displacement. As a result, they often fail to provide a comprehensive view of both local movement dynamics and global motion trajectories over time. This highlights the need for a more complete pose forecasting metric that can jointly assess the error of predicted joint movements, as well as the overall realism and coherence of predicted human motion.

### 2.4. GCN and Transformer Hybrid Architectures in Related Fields

While significant progress has been made with Graph Convolutional Networks (GCNs) and Transformers individually, to the best of our knowledge, no prior work has successfully integrated these two architectures into a unified model specifically for the task of multi-person pose forecasting. This gap represents an opportunity for advancement, as combining the strengths of GCNs in capturing spatial dependencies and Transformers in modeling long-range temporal dynamics could lead to more robust and accurate predictions in complex, interaction-heavy scenarios. In this paper, we aim to bridge this gap by proposing GCN-Transformer, a novel model that leverages both GCN and Transformer architectures for multi-person pose forecasting, potentially setting a new standard in the field.

Although no previous work has applied a GCN-Transformer hybrid directly to multi-person pose forecasting, this combination has demonstrated considerable success across several related fields. These studies provide valuable insights into the benefits of integrating structured relational modeling with dynamic sequence modeling. In the following, we briefly review selected examples where GCN-Transformer hybrids have been effectively applied to tasks such as trajectory prediction [30,31], time series forecasting [32,33], and pose estimation [34,35]. For example, Li et al. in [30] proposed a Graph-Based Spatial Transformer for predicting multiple plausible future pedestrian trajectories, which models both human-to-human and human-to-scene interactions by integrating attention mechanisms within a graph structure. Additionally, they present a Memory Replay algorithm to improve the temporal consistency of predicted trajectories by smoothing the temporal dynamics. Similarly, Aydemir et al. in [31] proposed a novel approach for predicting trajectories in complex traffic scenes. By utilizing a dynamic-weight learning mechanism, the model adapts to each person’s state while maintaining a scene-centric representation to ensure efficient and accurate trajectory prediction for all individuals. The model leverages GCNs to capture spatial interactions between individuals and employs Transformer-based attention to model temporal dependencies.

GCN and Transformer architectures have also been successfully applied to time series forecasting, a task of predicting future time intervals based on historical data. For instance, Hu et al. in [32] introduced a GCN-Transformer model designed to handle complex spatiotemporal dependencies in EV-battery-swapping-station load forecasting. The model integrates Graph Convolutional Networks (GCNs) to capture spatial relationships between stations and a Transformer to model temporal dynamics, allowing it to manage both spatial and temporal information simultaneously. Similarly, Xiong et al. in [33] introduced a model for chaotic multivariate time series forecasting. The model utilizes a Dynamic Adaptive Graph Convolutional Network (DAGCN) to model spatial correlations across variables and applies multi-head attention from the Transformer to capture temporal relationships. This hybrid approach demonstrates the effective application of GCNs and Transformers in tasks that require managing complex nonlinear data, such as chaotic systems, showing strong interpretability and performance across benchmark datasets.

GCN and Transformer architectures have also been successfully applied to pose estimation, a task of detecting human joint positions from an image. For example, Zhai et al. in [34] proposed the Hop-wise GraphFormer (HGF) module, which groups joints by k-hop neighbors and applies a transformer-like attention mechanism to model joint synergies. Additionally, the Intragroup Joint Refinement (IJR) module refines joint features, particularly for peripheral joints, using prior limb information. Furthermore, Cheng et al. in [35] presents GTPose, a novel model combining Graph Convolutional Networks (GCNs) and Transformers to enhance 2D human pose estimation. The model uses multi-scale convolutional layers for initial feature extraction, followed by Transformers to model the spatial relationships between keypoints and image regions. To further refine predictions, a Graph Convolutional Network models the topological structure between keypoints, capturing the relationships between joints.

While prior works have combined GCNs and Transformers in tasks such as trajectory forecasting, time series prediction, and pose estimation, these models typically apply GCNs for spatial encoding followed by Transformers for temporal modeling in a sequential or stacked manner. In contrast, our architecture is structured as a modular pipeline that first models social contexts using a Spatial-GCN applied across all individuals in the scene. This shared context is then injected into per-person forecasting branches using query token fusion, allowing each branch to access global scene information alongside individual motion patterns. Additionally, our forecasting module jointly incorporates both Transformer-based attention mechanisms and Temporal GCNs, enabling the complementary modeling of long-range temporal dependencies and local graph-based dynamics. To our knowledge, no prior GCN-Transformer hybrid applies this architecture to multi-person pose forecasting with such explicit scene-person disentanglement and fusion.

## 3. Background of Graph Convolutional Networks and Transformers

In recent years, two of the most prominent architectures for tasks like pose forecasting have been Graph Convolutional Networks (GCNs) and Transformer architectures. To better understand their foundations and effectiveness, we will provide a formalized overview of these architectures. It is important to note that the following descriptions remain generalized relative to GCN and Transformer architectures and do not delve into their specific application to multi-person pose forecasting, as this has already been addressed in the Related Work Section.

### 3.1. Graph Convolutional Networks

Conventional Convolutional Neural Networks (CNNs) operate on grid-like data structures like images, while GCNs are designed to work with non-Euclidean data, such as graphs, which consist of nodes (vertices) and edges representing relationships between the nodes. A graph is formally defined as G=(V,E), where *V* is the set of nodes and *E* is the set of edges. The key challenge in GCNs is to propagate information between nodes to capture the spatial structure of the graph.

GCNs can be broadly categorized into spatial and spectral graph convolutions [36]. Spatial-GCNs aggregate information from neighboring nodes based on their local structure. This aggregation can be extended to k-hop neighbors, where the neighborhood expands to include nodes within k steps of the target node, as in [37]. Spectral GCNs, on the other hand, transform graph data into the spectral domain, using the graph’s Laplacian to perform convolutions, but these often encounter computational challenges due to the size of the graph kernel. A simplified version of spectral convolutions, proposed by Kipf and Welling in [38], utilizes a first-order approximation, which is widely adopted due to its computational efficiency.

The general form of a GCN layer can be represented as follows:

(1) $$H^{\left(l+1\right)}=\sigma \left(\tilde{A}\;H^{l}\;W^{l}\right)$$

where $H^{l}$ represents the feature matrix at layer *l*, $\tilde{A}$ is the normalized adjacency matrix, $W^{l}$ is the learnable weight matrix at layer *l*, and σ is an activation function like ReLU.

**Figure 1:**
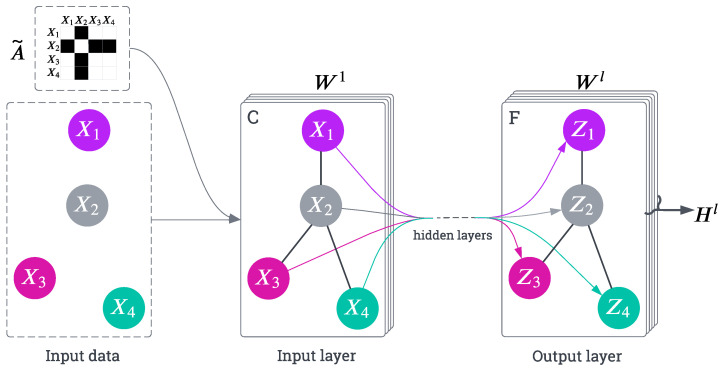
The figure depicts a multi-layer Graph Convolutional Network (GCN) architecture. The graph’s structure, defined by the normalized adjacency matrix $\tilde{A}$, is shared across all layers (edges shown as black lines). The input data (with *C* channels) are iteratively transformed at each layer *l* using $\tilde{A}$ and a learnable weight matrix $W^{l}$. The final layer outputs feature maps, *F*, capturing node relationships and properties through stacked graph convolutions.

Figure 1 illustrates the multi-layer GCN architecture, highlighting how the input features are progressively transformed through successive layers using the shared graph structure defined by the normalized adjacency matrix $\tilde{A}$. Traditionally, the adjacency matrix is predefined based on the structure of the graph (e.g., a human skeleton with fixed joint connections). However, in more advanced applications, especially in tasks like pose forecasting, the adjacency matrix $\tilde{A}$ can be treated as a learnable parameter [24,39], allowing the model to dynamically adapt the relationships between nodes (e.g., joints) based on the data. By making the adjacency matrix learnable, the network can adjust the strength or presence of connections between nodes, capturing more complex and data-driven relationships that may not be explicitly defined in the original graph. This is particularly useful for tasks involving non-static or flexible relationships, such as multi-person interactions or joint dynamics that change over time.

### 3.2. Transformer Architecture

The Transformer model, introduced by Vaswani in [40], has revolutionized the field of sequence modeling due to its effectiveness in capturing long-range dependencies and its parallel computation capabilities. Initially developed for natural language processing (NLP), where understanding contextual relationships between words across long sequences is essential, the Transformer architecture quickly surpassed traditional recurrent models such as LSTMs and GRUs. This success sparked widespread adoption across numerous domains, including computer vision, time-series forecasting, reinforcement learning, and human motion modeling.

Transformers rely on the attention mechanism that allows each element of the input sequence to interact with every other element. During processing, the attention mechanism assigns higher importance, or attention weights, to parts of the sequence that are most relevant for a given prediction or representation. This dynamic weighting enables the model to selectively focus on crucial inputs while diminishing the influence of less relevant ones, enhancing the ability to capture complex, long-range relationships without relying on sequential processing steps.

Because Transformers do not inherently model sequential order, they incorporate positional encodings into the input embeddings to preserve information about the position of each element within a sequence. These positional encodings can be predefined, typically using sine and cosine functions at varying frequencies [15,19,41,42], or learned as trainable parameters during model optimization [16,18]. By embedding positional information alongside content information, Transformers maintain the ability to reason about both the identity and the temporal order of elements, allowing them to capture complex sequential dependencies in various tasks.

Moreover, Transformers are inherently well suited for scenarios involving complex relational dynamics, a defining characteristic of sensor-based human motion data. Their global attention mechanism enables the model to dynamically prioritize the most relevant joints or individuals at each time step, allowing it to capture nuanced dependencies across space and time. This capability is particularly valuable in crowded or interaction-rich environments, where individual movements are not independent but influenced by the collective behavior of others in the scene.

At the core of the Transformer is the scaled dot-product attention, which computes the attention score as follows:

(2) $$\mathrm{Attention}\left(Q,K,V\right)=\mathrm{Softmax}\left(\frac{QK^{⊤}}{\sqrt{d_{k}}}\right)\;V$$

where *Q*, *K*, and *V* are the query, key, and value matrices derived from the input sequence, and $d_{k}$ is the dimensionality of the key vectors. The softmax function ensures that the attention weights sum up to one, enabling the model to focus on relevant parts of the sequence. The scaling factor $\sqrt{d_{k}}$ prevents the dot-product values from growing too large, which could cause vanishing gradients during backpropagation [40].

**Figure 2:**
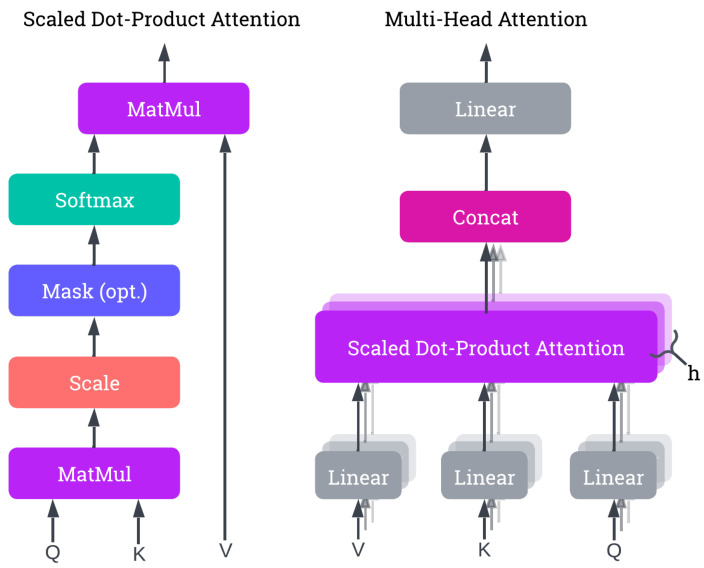
The figure illustrates the attention mechanism used in Transformer architecture. The left side depicts Scaled Dot-Product Attention, where the attention scores are computed using queries (*Q*), keys (*K*), and values (*V*), followed by scaling and a softmax operation. The right side shows Multi-Head Attention, consisting of multiple parallel Scaled Dot-Product Attention layers. The outputs of these parallel layers are concatenated and linearly transformed to produce the final attention output.

To enhance the model’s expressiveness, the Transformer uses multi-head attention, where multiple attention mechanisms run in parallel, and their outputs are concatenated:

(3) $$\mathrm{MultiHead}\left(Q,K,V\right)=\mathrm{Concat}\left({\mathrm{head}}_{1},\ldots ,{\mathrm{head}}_{h}\right)\;W^{O}$$

where ${\mathrm{head}}_{i}=\mathrm{Attention}\left(QW_{i}^{Q},KW_{i}^{K},VW_{i}^{V}\right)$, and $W_{i}^{Q}$, $W_{i}^{K}$, and $W_{i}^{V}$ are learnable weight matrices for the queries, keys, and values, respectively. The outputs are then transformed by a final weight matrix $W^{O}$ [40]. Figure 2 illustrates the calculations involved in the attention mechanisms of Transformers, including Scaled Dot-Product Attention and Multi-Head Attention, which aggregate multiple attention layers in parallel.

## 4. Problem Formulation for Multi-Person Forecasting

**Figure 3:**
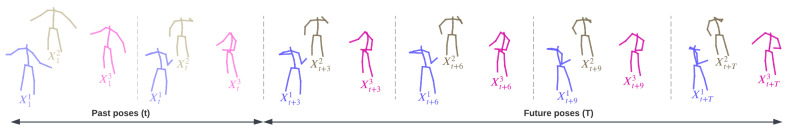
The figure illustrates the problem formulation for predicting the future movements of multiple individuals in a scene. Each individual is represented by joints (e.g., elbows, knees, shoulders), and the task is to forecast their trajectories over *T* time steps. The model receives historical pose sequences $X_{1:t}^{n}$ for each individual *n*, containing the positional data of joints in three-dimensional Cartesian coordinates. The objective is to predict future pose sequences $X_{t+1:T}^{n}$, extending *T* time steps into the future.

In the multi-person pose forecasting task, the aim is to forecast the forthcoming movements of multiple individuals within a given scene. Each individual in the scene is characterized by anatomical joints, typically including key areas such as elbows, knees, and shoulders. The task involves predicting the trajectories of these joints over a specified duration into the future, usually denoted by *T* time steps. To accomplish this predictive task, the model is provided with a sequence of historical poses for each individual. These historical poses encapsulate the positional information of each joint in three-dimensional Cartesian coordinates framed within a global coordinate system. This representation is standard in the field, as it reflects the native output of motion capture systems and 3D pose estimation models, and it allows for the straightforward computation of spatial relationships such as distances and velocities. For any given individual n=1…N, each historical pose is represented by a vector of *J* dimensions, where *J* signifies the number of tracked joints. Consequently, the entire historical sequence for individual *n* is represented as $X_{1:t}^{n}$, capturing the temporal evolution of poses up to the present moment. The length of the input pose sequence, denoted as *t*, dictates the number of historical poses the model uses for prediction. The index *n* ranges from 1 to *N*, where *N* corresponds to the total number of individuals observed within the scene. At its core, the model’s primary objective is to generate future pose sequences for each individual, denoted as $X_{t+1:T}^{n}$. Here, *T* reflects the future number of time steps that the model is tasked with forecasting. The problem’s formulation is graphically shown in Figure 3.

## 5. Proposed Architecture and Model

This paper proposes GCN-Transformer, a novel model for multi-person pose forecasting that emphasizes capturing complex interactions and dependencies between individuals within a scene. GCN-Transformer takes sequences of poses from all individuals in the scene as inputs, which are firstly preprocessed to enhance the data’s richness. These sequences are then processed through the Scene Module, which is designed to capture the interactions and dependencies between individuals within the scene. Following this, the Spatiotemporal Attention Forecasting Module combines this contextual information with each individual’s sequence to predict future poses. The following sections provide a detailed description of each component in the model’s architecture.

The architecture of GCN-Transformer is guided by complementary theoretical principles from graph-based and attention-based modeling. Graph Convolutional Networks (GCNs) are well suited for capturing structured spatial relationships, such as the physical dependencies among joints and the social connections between individuals in a shared scene. These structures act as relational inductive biases that help the model reason over pose and proximity with minimal supervision. In contrast, Transformers are powerful tools for modeling long-range temporal dependencies and contextual interactions. Their self-attention mechanism allows for the dynamic weighting of information across time and between individuals, without requiring sequential computation. By combining GCNs and Transformers, GCN-Transformer is able to model both local and global dynamics, capturing individuals’ joint relationships and interactions with temporal dependencies in multi-person scenes.

GCN-Transformer comprises two main modules: the Scene Module and Spatiotemporal Attention Forecasting Module. Initially, the input sequences, $X^{n\ldots N}$, are padded with the last known pose’s *T* times and augmented by incorporating their temporal differentiation, resulting in enriched sequences denoted as $Z^{n\ldots N}$. Temporal differentiation refers to the process of computing the difference between joint positions across consecutive time steps to obtain motion velocity or first-order dynamics. Formally, for each person *n*, we compute $\Delta X_{t}^{n}=X_{t+1}^{n}-X_{t}^{n}$, and we concatenate this velocity signal with the original sequence along the joint feature’s dimension. A zero-initialized frame is prepended to maintain temporal alignment. This results in a richer representation capturing both position and motion. These enriched sequences are concatenated and fed into the Scene Module. Within the Scene Module, a Spatiotemporal Fully Connected module encodes the poses into an embedding space. Subsequently, the output undergoes processing through the Spatial-GCN network designed to extract social features and dependencies. The resulting output *S* from the Scene Module is then forwarded into the Spatiotemporal Attention Forecasting Module for each *n*-th sequence $Z^{n}$, along with a query token $Q^{n}$ generated through one-hot encoding based on the position of the *n*-th sequence within the scene.

In the Spatiotemporal Attention Forecasting Module, the sequence $Z^{n}$ is encoded into the embedding space using a Spatiotemporal Fully Connected module (STFC). The resulting output is then concatenated with the extracted features *S* from the Scene Module and the query token $Q^{n}$ to create $W^{n}$. This fusion combines individual motion, scene-level context, and identity-specific signal. $W^{n}=\left[\mathrm{STFC}\left(Z^{n}\right);S;Q^{n}\right]$, where $\mathrm{STFC}\left(Z^{n}\right)\in R^{T\times d}$, $S\in R^{T\times d}$, and $Q^{n}\in R^{1\times d}$ (broadcasted across *T*). Subsequently, $W^{n}$ is simultaneously passed into the Spatiotemporal Transformer Decoder and Temporal-GCN modules. The outputs from both modules are concatenated and processed through a Spatiotemporal Fully Connected module to generate the final prediction ${\hat{y}}^{n}$.

**Figure 4:**
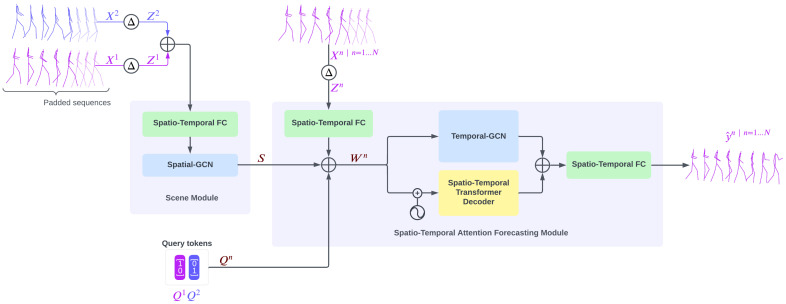
The figure depicts the architecture of the GCN-Transformer model. In the preprocessing step, input sequences $X^{1}$ and $X^{2}$ are padded with the last pose to match the full length of the sequence, and they are enriched with their temporal differentiation Δ, resulting in sequences $Z^{1}$ and $Z^{2}$. These sequences are then jointly processed by the Scene Module to extract social features and dependencies, producing the output *S*. Finally, to produce the final predictions, the output *S* is subsequently fed into the Spatiotemporal Attention Forecasting Module for each *n*-th sequence $Z^{n}$, along with a query token $Q^{n}$ generated via one-hot encoding based on the position of the *n*-th sequence within the scene.

The architecture of GCN-Transformer is shown in Figure 4, and the full forward pass of GCN-Transformer is outlined in Algorithm 1.
**Algorithm 1:** Pseudocode outlining the end-to-end forward pass of GCN-Transformer. The model first applies temporal differentiation to augment pose sequences for all individuals in the scene. These enriched sequences are embedded and passed through a Spatial GCN to extract scene-level context. Each individual’s sequence is then fused with the scene context and an identity-specific query token before being processed in parallel by a Spatiotemporal Transformer Decoder and a Temporal GCN. The outputs are concatenated and passed through a final Spatiotemporal Fully Connected module to produce future pose predictions.**Input**: Pose sequences $X_{1:t}^{1\ldots N}$ for *N* individuals, each with *J* joints in 3D space**Output**: Predicted future pose sequences ${\hat{Y}}_{1:t+T}^{1\ldots N}$
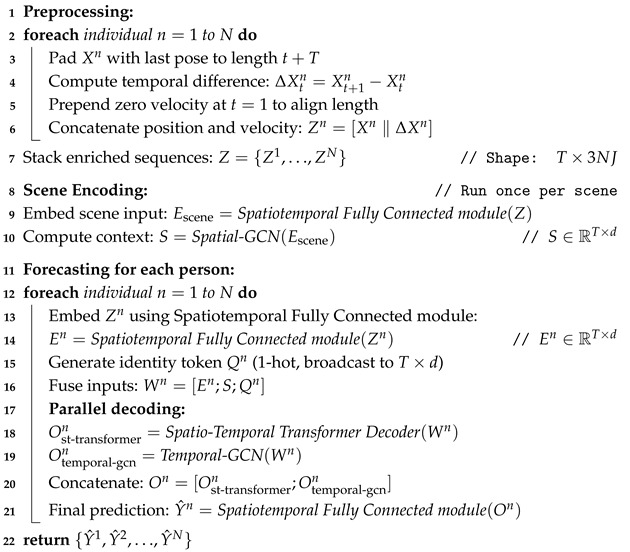


### 5.1. Spatiotemporal Fully Connected Module

The Spatiotemporal Fully Connected module is a lightweight component that projects pose sequences into a higher-dimensional embedding space, making them suitable for processing by downstream modules. It consists of two fully connected layers that independently process the spatial and temporal dimensions of the input. Given an input sequence $X\in R^{T\times 3NJ}$, where *T* is the number of time steps, *N* is the number of individuals, *J* is the number of joints, and each joint is represented in 3D Cartesian space. The first fully connected layer operates along the spatial dimension, and it maps each frame-level pose vector of dimension 3NJ to a higher-dimensional representation, resulting in an intermediate output of shape $R^{T\times d}$. Subsequently, a second fully connected layer is applied across the temporal dimension, allowing the model to capture short-term temporal patterns and refine the sequence-level encoding. The final output remains in $R^{T\times d}$ and serves as the input to both the Scene Module and Spatiotemporal Attention Forecasting Module, where it is further processed by GCN and Transformer components.

### 5.2. Scene Module

The Scene Module is designed to enhance input data representation by leveraging temporal and spatial information. It comprises two key elements: a Spatiotemporal Fully Connected module and the Spatial-GCN. The Spatiotemporal Fully Connected module serves as an initial processing unit, transforming the enriched input sequence $Z^{n\ldots N}$ into a higher-dimensional embedding space, refining the input data and preparing them for subsequent modules through spatial and temporal transformations. In conjunction with the Spatiotemporal Fully Connected module, the Spatial-GCN module serves to uncover intricate patterns embedded within the data, specifically focusing on extracting interaction dependencies and dynamics among individuals within the scene. Comprising eight GCN blocks with learnable adjacency matrices, this module employs various techniques, including batch normalization, dropout, and Tanh activation functions, to enhance feature extraction and maintain the integrity of the structural information present in the input data. To further enhance the model’s ability to capture social dependencies and maintain realistic spatial relationships between joints of the people in the scene, we compute the inter-individual joint distance loss on the output *S*.

### 5.3. Spatiotemporal Attention Forecasting Module

The Spatiotemporal Attention Forecasting Module predicts future poses by synthesizing information from various sources, including the input sequence $Z^{n}$, scene context *S*, and positional query token $Q^{n}$ associated with sequence $Z^{n}$. Initially, the input sequence $Z^{n}$ undergoes encoding via the Spatiotemporal Fully Connected module, transforming into an embedded space. Subsequently, this encoded sequence is concatenated with the scene context *S* and the positional query token $Q^{n}$ to form $W^{n}$. This composite representation $W^{n}$ undergoes parallel processing through two key components: the Spatiotemporal Transformer Decoder and the Temporal-GCN modules.

The Spatiotemporal Transformer Decoder comprises two attention blocks positioned after the learnable positional encoding of $W^{n}$. The first attention block is followed by fully connected layers that operate on the spatial dimension, facilitating the extraction of spatial features. Conversely, the second attention block is followed by Temporal Convolutional Network (TCN) layers, which specialize in capturing long-term temporal dependencies and temporal patterns within the data. Concurrently, the Temporal-GCN module, composed of eight GCN blocks with learnable adjacency matrices, operates on $W^{n}$ to extract and refine temporal dependencies, thereby enhancing the temporal representation separate from the Spatiotemporal Transformer Decoder.

Finally, the Spatiotemporal Attention Forecasting Module integrates the extracted features using Spatiotemporal Fully Connected module, resulting in the generation of the final pose sequence prediction ${\hat{y}}^{n}$. This fusion process ensures that the module leverages the diverse information captured across spatial, temporal, and contextual dimensions to produce accurate and reliable predictions for future poses.

### 5.4. Data Preprocessing

We opted against employing any data preprocessing techniques for our model; instead, we utilized raw data from the datasets. This approach was chosen to compel the model to learn the intricate structure of the human skeleton and the dynamic nature of movement. Conventional preprocessing methods, such as employing Discrete Cosine Transform (DCT) to encode Cartesian coordinates into frequencies, often yield poses that appear ghost-like and lack the nuanced dynamics of human movement, like in [13,15,16,17]. Moreover, techniques like predicting temporal differentiation that is subsequently added to the last known pose to generate the final result can produce invalid poses over the long term due to the model’s lack of awareness regarding human structural information, like in [12,15,18,19,20].

### 5.5. Data Augmentation

Data augmentation is used for enhancing the robustness and generalization capability of pose forecasting models. Building upon methods utilized in [17], we extended the augmentation strategy with new methods to introduce further variations in the training data. Inspired by [17], we adopted several effective methods: sequence reversal, which reverses the temporal order of input sequences to expose the model to diverse temporal patterns; random person permutation, which shuffles the order of individuals within a scene to accommodate different person arrangements and interactions; random scaling, which introduces variations in pose scale to simulate varying heights of the people; random orientation, where poses are randomly rotated to simulate different camera viewpoints or human orientations; and random positioning, which shifts the positions of individuals within the scene to introduce spatial variability.

Expanding upon these methods, we introduced new techniques to enrich the dataset further. One method involved randomizing the joint order of individuals in a scene, encouraging the model to learn complex skeleton representations and adapt to different joint configurations. Additionally, we used a method to randomize the XYZ axes of individuals, enhancing pose variation by altering the orientation and positioning of poses in 3D space. Lastly, we varied the dataset’s sampling frequency, using frequencies 1–4 to capture slower and faster sequences, though this type of sampling is performed during the preprocessing step.

All augmentations, except for sampling frequencies, are applied dynamically to each sampled batch of scene sequences during training. Each augmentation method is applied with a specific probability, introducing controlled variability into the training data. For instance, sequence reversal, random person permutation, random scaling, and random positioning each have a 50% probability of being applied, while random orientation, random joint order, and random XYZ-axis order are applied with a 25% probability. Furthermore, there is a 25% probability that no augmentation will be applied to a given sequence, ensuring that the model is exposed to both augmented and unaugmented data. These augmented datasets enable the model to learn robust features and adapt effectively to diverse scenarios, improving its performance and generalization capability in pose forecasting tasks.

We progressively introduced each method during development and empirically observed consistent reductions in training loss, indicating improved learning dynamics. All augmentation strategies were designed to preserve structural validity, and none produced implausible or invalid pose sequences. Importantly, all augmentations in our pipeline are applied consistently across the entire scene, meaning that the same transformation is applied to all individuals’ pose sequences within a given scene to ensure that augmented motions remain coherent and socially consistent. Furthermore, since each augmentation process is applied with controlled probability and independently of others, we found no clear evidence of conflicting interactions or degradation in data quality. In practice, the combined use of all proposed augmentations led to the most effective training results across all datasets, as we also show in the ablation study (Section 7).

### 5.6. Training

Our model optimizes its parameters by minimizing the error between the predicted and ground truth poses, using a loss commonly referred to as reconstruction loss (REC). This is a standard approach in pose forecasting and is widely adopted in prior work due to its simplicity and direct correlation with spatial prediction accuracy. REC is typically computed as the L2 distance between corresponding joints in the predicted and ground truth sequences, ensuring that the forecasted poses remain close to the true positions frame by frame.

However, while REC provides a useful baseline for learning pose positions, it has several limitations, particularly in the context of multi-person and dynamic motion forecasting. REC measures pose similarity on a per-joint, per-frame basis, and as such, it does not account for the temporal continuity of movements or the relational dynamics between individuals. This can lead to predicted sequences that are spatially accurate in isolated frames but lack smoothness over time or consistency in movement dynamics. For instance, a model trained with REC alone may generate plausible individual poses that result in jittery motion or unrealistic group behavior, such as individuals moving without regard for nearby participants.

To address these shortcomings, we introduce two additional loss terms that target complementary aspects of human motion. First, the multi-person joint distance (MPJD) loss enhances the model’s ability to capture social and spatial interactions by penalizing discrepancies in joint distances between individuals across time. This encourages the Scene Module to improve model interaction dependencies and produce socially coherent pose sequences. Second, we incorporate a Velocity loss (VL), which prioritizes the learning of consistent temporal dynamics. By penalizing deviations in joint velocities between predicted and ground truth sequences, the VL term helps the model generate smoother and more realistic motion trajectories, reducing jitter and improving the fluidity of movement. The effectiveness of both additional losses is demonstrated in the ablation study (Section 7).

The final loss function is determined by combining the standard reconstruction loss with an additional multi-person joint distance loss (MPJD), scaled by a factor denoted as γ, used to adjust the effect of the MPJD loss on the overall loss. Both the output and scene predictions are subjected to Velocity Loss (VL), with Velocity Loss for the output from the Scene Module also scaled by the γ factor. To measure the error between the predicted and ground truth coordinates, we employ $L_{2}$-norm loss, aiming to minimize this error during training.

The final loss is calculated as follows:

(4) $$L_{\mathrm{REC}}=\frac{1}{N}\sum_{n=1}^{N}||{{\hat{y}}_{n}-y_{n}||}_{2}$$

(5) $$L_{\mathrm{MPJD}}=\frac{1}{N(N\;-\;1)}\sum_{n=1}^{N}\sum_{p=1}^{N}||{\left({\hat{y}}_{n}-{\hat{y}}_{p}\right)-\left(y_{n}-y_{p}\right)||}_{2}$$

(6) $$L_{\mathrm{REC}\_\mathrm{VL}}=\frac{1}{N}\sum_{n=1}^{N}||{\Delta {\hat{y}}_{n}-\Delta y_{n}||}_{2}$$

(7) $$L_{\mathrm{MPJD}\_\mathrm{VL}}=\frac{1}{N(N-1)}\sum_{n=1}^{N}\sum_{p=1}^{N}||{\Delta {\hat{d}}_{n,p}-\Delta d_{n,p}||}_{2}$$

(8) $$L=L_{\mathrm{REC}}+L_{\mathrm{REC}\_\mathrm{VL}}+L_{\mathrm{MPJD}}\times \gamma +L_{\mathrm{REC}\_\mathrm{VL}}\times \gamma$$

where *N* represents the number of people in the scene; ${\hat{y}}_{n}$ and ${\hat{y}}_{p}$ represent the predicted pose sequence of the *n*-th and *p*-th person in the scene, while $y_{n}$ and $y_{p}$ represent the corresponding ground truth pose sequence of *n*-th and *p*-th person in the scene. ${||\cdot ||}_{2}$ denotes the Euclidean distance (L2 norm), and $\frac{1}{N}\sum_{n=1}^{N}$ represents the mean distance across all people in the scene. The Δ represents temporal differentiation, where $\Delta y_{n}=y_{n}^{t}-y_{n}^{t+1}$ for t=0,1,…,T−1 and $\Delta {\hat{y}}_{n}={\hat{y}}_{n}^{t}-{\hat{y}}_{n}^{t+1}$ for t=0,1,…,T−1. The predicted velocities of joint distances between individuals are represented with $\Delta {\hat{d}}_{n,p}$, while $\Delta d_{n,p}$ represents the ground truth velocities of joint distances between individuals.

Including MPJD and VL losses in the training process significantly enhances the practical applicability of multi-person pose forecasting models in real-world scenarios. The MPJD loss encourages the model to learn interaction dynamics between individuals in a scene, helping it capture how one individual’s movements influence others. This is particularly useful in scenarios such as crowd monitoring, group behavioral analysis, and human–robot collaboration, where understanding interpersonal interactions is essential. On the other hand, the VL loss emphasizes temporal velocities between subsequent poses, promoting the generation of fluid and natural motion sequences. This is crucial in applications like animation, virtual reality, and autonomous systems, where smooth and realistic motion transitions are essential. Together, these losses address the challenges of producing rigid or disconnected poses, ensuring that the model generates dynamic, context-aware predictions.

We trained our model for 512 epochs with a batch size of 256, which was the largest manageable size given our hardware constraints. The extended training duration was chosen to accommodate the strong and dynamic augmentation strategy, which introduced extensive variability to the data, necessitating longer training for the model to effectively learn from these variations. Observing that the performance improvements plateaued at around 512 epochs, we determined that this duration was sufficient for optimal convergence. The Adam optimizer, a standard choice in pose forecasting, was chosen due to its adaptability and efficiency in handling complex, dynamic loss landscapes, especially with the strong augmentations applied. After testing multiple learning rates, we set an initial learning rate of 0.001, finding that it balanced effective learning with stability. A higher learning rate caused the loss to oscillate heavily, likely due to abrupt shifts in the solution space introduced by the strong augmentation, and in some cases, gradients would explode. To guide the model closer to the optimal solution, we reduced the learning rate to 0.0001 after 256 epochs, ensuring smoother convergence in the later stages of training. We also carefully tuned the γ parameter, which scales the MPJD loss, by analyzing values from 0 to 1. A value of 0.1 was selected, as it provided the best balance in guiding the model to capture both spatial dependencies and movement dynamics effectively.

## 6. Experimental Results

In our experimental evaluation of the GCN-Transformer, we employed four distinct datasets: CMU-Mocap, MuPoTS-3D, SoMoF, and ExPI. To assess the model’s performance, we define evaluation metrics that quantify the error between predicted poses and ground truth. Through comprehensive analysis, we evaluated our model’s performance on all datasets and conducted a comparative study against state-of-the-art models in the domain of multi-person pose forecasting. All models used for the experimental results were retrained from scratch using their official implementations, with the exception of Future Motion, which we re-implemented based on the details provided in the original paper. We followed the reported training protocols and hyperparameters wherever available and performed validation-based tuning only for Future Motion due to missing implementation details. All models were trained and evaluated under a consistent experimental setup to ensure a fair and meaningful comparison with our proposed method.

### 6.1. Metrics

The MPJPE (Mean Per Joint Position Error) is a commonly used metric for evaluating the performance of pose forecasting methods [15,16,17,18,43]. It measures the average Euclidean distance between the predicted joint positions and the corresponding ground truth positions across all joints. The lower the MPJPE value, the closer the predicted poses align with the ground truth. This metric provides a joint-level assessment of pose forecasting performance. The MPJPE metric is calculated as follows:

(9) $$E_{\mathrm{MPJPE}}\left(\hat{y},y,\varphi \right)=\frac{1}{J_{\varphi }}\sum_{j=1}^{J_{\varphi }}||{P_{\hat{y},\varphi }^{\left(f\right)}\left(j\right)-P_{y,\varphi }^{\left(f\right)}\left(j\right)||}_{2}$$

where *f* denotes a time step, and φ denotes the corresponding skeleton. $P_{\hat{y},\varphi }^{\left(f\right)}\left(j\right)$ is the estimated position of joint *j*, and $P_{y,\varphi }^{\left(f\right)}\left(j\right)$ is the corresponding ground truth position. $J_{\varphi }$ represents the number of joints. ${||\cdot ||}_{2}$ denotes the Euclidean distance (L2 norm), and $\frac{1}{J_{\varphi }}\sum_{j=1}^{J_{\varphi }}$ represents the mean distance across all joints.

Another commonly employed metric in pose forecasting evaluation is the Visibility-Ignored Metric (VIM), initially proposed by Adeli et al. in [22]. The VIM is computed by assessing the mean distance between the predicted and ground truth joint positions at the last pose *T*. This calculation involves flattening the joint positions and coordinates dimensions into a unified vector representation, resulting in a vector dimensionality of 3J, where *J* denotes the number of joints. Subsequently, the Euclidean distance (L2 norm) is computed between the corresponding ground truth and predicted joint positions. The average distance across all joints yields the final VIM score. The SoMoF Benchmark adopts this metric for its evaluation framework. The VIM metric computation can be expressed as follows:

(10) $$E_{\mathrm{VIM}}\left(\hat{y},y,\varphi \right)=\frac{1}{3J_{\varphi }}\sum_{j=1}^{3J_{\varphi }}||{P_{\hat{y},\varphi }^{\left(j\right)}-P_{y,\varphi }^{\left(j\right)}||}_{2}$$

where *J* represents the number of joints, $P_{y,\varphi }^{\left(i\right)}$ is the ground truth position of the i-th joint (flattened), $P_{\hat{y},\varphi }^{\left(i\right)}$ is the predicted position of the i-th joint (flattened), ${||\cdot ||}_{2}$ denotes the Euclidean distance (L2 norm), and $\frac{1}{3J_{\varphi }}\sum_{j=1}^{3J_{\varphi }}$ represents the mean distance across all joints.

### 6.2. Datasets

We employed distinct datasets for both training and evaluation, aligning with the methodology of previous models such as SoMoFormer [16], MRT [15], MPFSIR [17], and JRTransformer [18]. For training, we utilized the 3D Poses in the Wild (3DPW) [44] and Archive of Motion Capture As Surface Shapes (AMASS) [45] datasets. The 3DPW dataset contains over 60 video sequences containing scenes with two individuals, capturing human motion in real-world scenarios, including accurate reference 3D poses in natural scenes, such as people shopping in the city, having coffee, or playing sports, recorded with a moving hand-held camera. The dataset was collected using a combination of vision-based sensors and inertial measurement units (IMUs), which provided high-fidelity motion tracking in unconstrained environments. To adhere to the evaluation protocol of the SoMoF benchmark [22], we employed a specific split of the 3DPW dataset, where the train and test sets are inverted. Thus, we trained all models on the 3DPW test set and subsequently evaluated them on the 3DPW train set. This inversion was originally introduced by the authors of the SoMoF benchmark [22] due to the preprocessing of the 3DPW dataset, which created a larger number of sequences in the test set than in the training set, thus inverting the datasets allowed for a more robust training set. By following this protocol, we ensure that our results are directly comparable with other multi-person pose forecasting models evaluated under the same conditions. Specifically, for the SoMoF test set, data from the original 3DPW training set were sampled without overlap, producing distinct pose sequences. In contrast, the SoMoF training set was generated by sampling the original 3DPW testing set with overlap, employing a sliding window of 1 to capture a broader range of pose variations. The validation set remained consistent with the original 3DPW dataset, which was sampled without overlap.

On the other hand, the AMASS dataset provides an extensive collection of human motion capture sequences, totaling over 40 h of motion data and 11,000 motions represented as SMPL mesh models. AMASS unifies multiple optical marker-based motion capture datasets within a common framework, where motion data were originally collected using high-precision marker-based tracking systems. During the training process, we utilized the CMU, BMLMovi, and BMLRub subsets of the AMASS dataset, which provided a diverse and large-scale dataset. Given that many sequences within this dataset are single-person, we employed a technique to synthesize additional training data by combining sampled sequences to generate multi-person training data.

In contrast to recent works [15,16,17,18,19] that utilize the SoMoF Benchmark [22] alongside the Carnegie Mellon University Motion Capture Database (CMU-Mocap) [46] and the Multi-person Pose Estimation Test Set in 3D (MuPoTS-3D) [47] for model evaluation, our study additionally presents results on the Extreme Pose Interaction (ExPI) [48] dataset.

The CMU-Mocap and MuPoTS-3D datasets contain scenes with three individuals, with approximately 8000 annotated frames of poses across 20 real-world scenes. However, the movements captured are primarily simplistic, with limited interactions, often resulting in sequences where individuals maintain largely static poses or perform minimal motions. While we include evaluations on CMU-Mocap and MuPoTS-3D to ensure completeness and facilitate comparison with prior works, we emphasize that models trained or evaluated on these datasets may struggle to demonstrate their full capabilities in forecasting socially coherent, dynamic multi-person motion.

Therefore, after presenting initial results on CMU-Mocap and MuPoTS-3D, we focus our full analysis on the SoMoF Benchmark and the Extreme Pose Interaction (ExPI) dataset, both of which feature two-person scenes but offer significantly more challenging and realistic multi-person motion scenarios. In particular, ExPI contains dynamic sequences involving two couples engaged in physically demanding and interaction-heavy activities. The dataset was collected using a multi-sensor motion capture system comprising 68 synchronized and calibrated RGB cameras, along with a high-resolution infrared-based motion capture setup featuring 20 infrared mocap cameras. This comprehensive setup makes ExPI particularly well suited for evaluating complex, coordinated multi-person interactions in controlled yet naturalistic settings.

### 6.3. Results on CMU-Mocap and MuPoTS-3D

We first evaluate the GCN-Transformer against several state-of-the-art (SOTA) multi-person pose forecasting models, including MRT [15], Future Motion [13], SoMoFormer [16], JRTransformer [18], LTD [3], and MPFSIR [17]. Following established protocols, we trained all models using a synthesized dataset created by combining sampled motions from the CMU-Mocap database to simulate three-person interaction scenes. Evaluations were conducted on both test sets from the CMU-Mocap and MuPoTS-3D datasets.

For the Carnegie Mellon University Motion Capture Database (CMU-Mocap) [46], we adopt the training and testing splits provided by Wang et al. in [15]. Specifically, the dataset’s construction involves combining two-person motion sequences with an additional randomly sampled third individual, introducing a degree of randomness into the generated scenes. To ensure fairness, the same generated datasets are used across all evaluated models.

Each input sequence consists of 15 historical frames (corresponding to 1000 ms), and the models are tasked with forecasting the subsequent 45 frames (3000 ms into the future). Each individual’s pose is annotated with 15 joints, provided both as inputs and as ground truth for evaluation. We assessed performance using the Mean Per Joint Position Error (MPJPE) metric, which is reported at 1, 2, and 3 s into the future to align with evaluation from [15]. All models are retrained and evaluated under identical conditions using the official code and data released by [15].

As summarized in Table 1, the GCN-Transformer consistently outperforms all competing methods on both CMU-Mocap and MuPoTS-3D datasets, achieving new state-of-the-art performance in these settings.

The results demonstrate that the proposed GCN-Transformer consistently outperforms all competing models across both the CMU-Mocap and MuPoTS-3D test sets. These improvements are observed consistently across short-term and long-term forecasting horizons, indicating the model’s strong ability to maintain prediction performance even as the forecast extends further into the future. Among the baselines, MPFSIR, JRTransformer, and LTD perform relatively competitively but still lag behind GCN-Transformer at all evaluation points. Interestingly, the model LTD, designed for single-person forecasting, performs relatively well given its lack of explicit multi-person modeling capabilities. In contrast, models such as MRT, SoMoFormer, and Future Motion show substantially higher errors, particularly as the forecast horizon increases, suggesting weaker mechanisms for modeling long-term temporal dependencies in multi-person settings. It is also noteworthy that the ordering of model performance shifts between the CMU-Mocap and MuPoTS-3D datasets. This variability indicates that many models are sensitive to the specific characteristics of the dataset and highlights a lack of consistent generalization ability across different multi-person forecasting environments.

The strong results achieved by the GCN-Transformer highlight its ability to forecast complex multi-person motion accurately over both short and long time horizons. Its consistent improvements across different datasets demonstrate robustness and generalization. These findings validate the importance of combining spatial and temporal reasoning for multi-person forecasting tasks. In the following sections, we further evaluate GCN-Transformer on more socially complex datasets (SoMoF and ExPI) to assess its performance in even more dynamic and challenging scenarios.

### 6.4. Results on SoMoF Benchmark

The SoMoF Benchmark, introduced by Adeli et al. in [22], serves as a standardized assessment platform for evaluating the performance of multi-person pose forecasting models. The SoMoF Benchmark is derived from the 3DPW dataset, where every other frame is sampled to lower the original frames per second (FPS) from 30 to 15. This benchmark task involves predicting the subsequent 14 frames (equivalent to 930 milliseconds) based on 16 frames (1070 milliseconds) of preceding input data, encompassing joint positions for multiple individuals. The evaluation uses the Visibility-Ignored Metric (VIM), measuring performances across various future time steps. Similarly to [13,16,17,18], all evaluated models in this paper were trained to utilize data from the 3DPW [44] and AMASS [45] datasets. During training, emphasis was placed solely on the 13 joints evaluated within the SoMoF framework. To ensure fairness in the comparisons, a practice observed in various studies such as [18,19,20] was adopted, whereby the final results are reported based on the epoch with the lowest average VIM score on the test dataset. Furthermore, problem formulation remained consistent for all evaluated models, focusing on predicting the next 14 frames using 16 input data frames. This differs from methodologies advocated by [13,16,18] to divide formulations into two separate problem formulations for short-term and long-term optimization, which inherently enhances the model’s performance.

We conducted a comparative analysis of evaluated methods on the SoMoF Benchmark test set, as presented in Table 2, demonstrating that our model consistently achieves state-of-the-art results compared to competing models.

The results demonstrate the superior performance of the proposed GCN-Transformer across both VIM and MPJPE metrics, establishing it as a state-of-the-art solution in multi-person pose forecasting. While SoMoFormer emerges as a formidable competitor, particularly in long-term forecasting, GCN-Transformer consistently outperforms all models, especially when considering the overall metric, which aggregates performance across all evaluated time intervals. Interestingly, despite the reported similar performance to SoMoFormer, the JRTransformer fails to achieve competitive results in this evaluation. Conversely, the Future Motion model, introduced in 2021, demonstrates commendable performance, rivaling even the most recent state-of-the-art models. The MPFSIR model is not far off either, achieving this performance with only a fraction of parameters compared to others. Finally, the GCN-Transformer* showcases significantly superior results owing to its training with an integrated validation dataset. This variant currently leads the official SoMoF Benchmark leaderboard at https://somof.stanford.edu.

**Figure 5:**
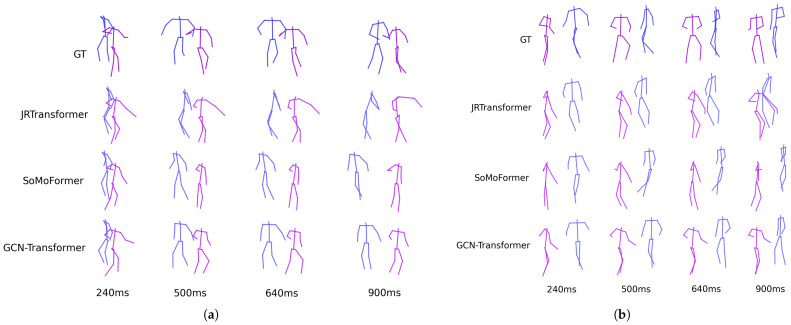
The figure displays predicted poses on two example sequences from the SoMoF Benchmark test set for the best-performing models: JRTransformer, SoMoFormer, and GCN-Transformer, with GT representing the ground truth. Sequence (**a**) shows two people rotating around each other, while sequence (**b**) shows two people meeting and then walking together in the same direction. The visual comparison reveals that while JRTransformer and SoMoFormer struggle to create valid poses, the GCN-Transformer generates both valid poses and realistic movement.

Figure 5 shows the predicted poses for two sequences from the SoMoF Benchmark test set, comparing the performance of the best-performing models, JRTransformer, SoMoFormer, and GCN-Transformer, with the ground truth (GT) also displayed for comparison. The figures reveal that both JRTransformer and SoMoFormer encounter difficulties in generating valid poses, often producing unrealistic joint configurations and movements. In contrast, the GCN-Transformer model demonstrates a clear advantage, consistently generating valid poses and realistic movements.

### 6.5. Results on ExPI Dataset

The Extreme Pose Interaction (ExPI) dataset, described in [48], features two pairs of dancers engaging in 16 distinct extreme actions. These actions include aerial maneuvers, with the first seven being performed by both dancer couples. Subsequently, six additional aerials are executed by Couple 1, while the remaining three are carried out by Couple 2. Each action is repeated five times to capture variability, resulting in a collection of 115 sequences recorded at 25 frames per second (FPS) and 60,000 annotated 3D body poses.

Taking inspiration from the data partitioning outlined in [48], we designate all actions executed by Couple 2 as the training set and those performed by Couple 1 as the test set. This approach deviates slightly from the dataset’s division presented by Guo et al. in [48], as we incorporate common actions performed by both couples and actions performed exclusively by one couple into the training set. This dataset split emulates both the Common action split and Unseen action split described in [48], consolidating them into a single split.

We employ a sliding-window technique with overlapping sequences to sample the training data, whereas the testing data are sampled sequentially without overlaps. Additionally, we downsample each sequence by selecting every other frame, reducing the original frames per second (FPS) from 25 to 12.5 FPS. Following the precedent set by the SoMoF Benchmark, we utilize 16 frames (equivalent to 1280 milliseconds) to predict the subsequent 14 frames (equivalent to 1080 milliseconds). Moreover, we apply a scaling factor of 0.39 to maintain consistency in person scale with the SoMoF Benchmark, the dataset on which the models are developed.

We conducted a comparative analysis of evaluated methods on the ExPI test set, as presented in Table 3, demonstrating that our model consistently achieves state-of-the-art results compared to competing models. The results on the ExPI dataset differ significantly from those on the SoMoF Benchmark dataset, revealing notable performance degradation in some of the previously strong models. SoMoFormer, a close competitor on the SoMoF Benchmark, performs substantially worse on the ExPI dataset, surpassed by JRTransformer and MPFSIR. This drop in performance highlights the model’s sensitivity to different dataset characteristics. Similarly, the Future Motion model, which had proven to be a strong contender on the SoMoF Benchmark, is now outperformed by almost all other models. This indicates that the Future Motion model’s performance is heavily influenced by the dataset’s characteristics, showcasing its lack of robustness across diverse data scenarios. Interestingly, JRTransformer, which was not as competitive on the SoMoF Benchmark, emerges as a close competitor to GCN-Transformer on the ExPI dataset. Despite this, the proposed GCN-Transformer remains the clear winner across all time intervals, reaffirming its superior performance and generalizability.

**Figure 6:**
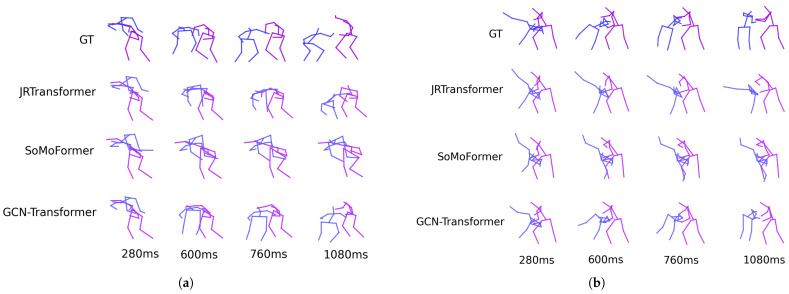
The figure displays predicted poses on two example sequences from the ExPI test set for the top-performing models, JRTransformer, SoMoFormer, and GCN-Transformer, with GT indicating the ground truth. Sequence (**a**) shows one person jumping off the shoulders of another, while sequence (**b**) shows one person performing a cartwheel assisted by another. The comparison illustrates that JRTransformer and SoMoFormer struggle with generating valid movements, often repeating the last known pose. In contrast, the GCN-Transformer demonstrates its capability to create realistic and dynamic movements.

Figure 6 shows the predicted poses for two sequences from the ExPI test set, showcasing the performance of the best-performing models, JRTransformer, SoMoFormer, and GCN-Transformer, with the ground truth (GT) also displayed for comparison. The results highlight a significant distinction in model performance. JRTransformer and SoMoFormer struggle to generate valid movements, often defaulting to repeating the last known pose rather than predicting dynamic and realistic trajectories. In contrast, the GCN-Transformer model maintains the integrity of the poses and successfully predicts realistic and coherent movement patterns.

### 6.6. Discussion of Comparative Advantages

While quantitative results establish the superior performance of our proposed GCN-Transformer model across all datasets, a deeper examination helps explain why it consistently outperforms prior approaches, particularly in interaction-heavy or socially complex scenarios. Methods such as MPFSIR and SoMoFormer primarily rely on dense fully connected layers or sequence-level attention, often treating individuals independently or relying on predefined assumptions about social structure. As a result, these models may struggle to encode fine-grained interaction dependencies or adapt to dynamically changing social configurations. In contrast, GCN-Transformer introduces a modular pipeline that combines learnable spatial reasoning (via the Spatial-GCN) with long-range temporal and spatial attention (via the Spatiotemporal Transformer Decoder), allowing it to reason jointly over the entire scene.

This design proves to be especially effective in datasets like ExPI, where highly coordinated motions (e.g., one person lifting or reacting to another) require the model to interpret subtle cues in one person’s movement that inform another’s. In these cases, baseline models often fail to capture the anticipatory or dependent nature of motion between individuals, producing disjointed or static predictions. We observe that GCN-Transformer maintains synchronization across subjects in such sequences and adapts more effectively to rapid transitions or uncommon poses, suggesting that its architectural integration of scene context and temporal dynamics enables stronger generalization.

Furthermore, the attention mechanisms in GCN-Transformer contribute to robustness in the presence of joint noise, as is sometimes the case in CMU-Mocap or MuPoTS-3D. Instead of relying uniformly on all joints or time steps, the model learns to attend selectively to informative joints and keyframes. This results in more stable predictions, even when input signals are imperfect, a scenario frequently encountered in real-world settings. Taken together, these architectural choices explain GCN-Transformer’s consistently strong performance across diverse motion types, social contexts, and temporal horizons.

To assess the generalization ability and performance consistency of the evaluated models, we compute the percentage improvement over the Zero-Velocity baseline across all four datasets, as summarized in Table 4. This analysis uses the “Overall” MPJPE values reported in the earlier result tables, which reflect the average prediction error across the entire forecasting horizon. The percentage improvement is calculated using the following formula: $\mathrm{Improvement}=\left(\mathrm{Zero}\;\mathrm{Velocity}-\mathrm{Method}\right)/\mathrm{Zero}\;\mathrm{Velocity}\times 100\%$. We use the Zero-Velocity model as a consistent reference point because it represents the most basic forecasting strategy, where the model simply repeats the last observed pose. Comparing raw MPJPE values across datasets is often not meaningful, as these values are strongly influenced by dataset-specific characteristics such as the amount of movement in the scenes, the difficulty of the motion patterns, and the prediction horizon. By instead reporting the improvement relative to the Zero-Velocity baseline, we obtain a normalized measure of model performance that enables more interpretable comparisons across different datasets.

For this analysis, we group the datasets into two categories based on the number of individuals in the scene and other shared characteristics. The CMU-Mocap and MuPoTS-3D datasets form a group of three-person scenes. These datasets both feature a three-second prediction horizon and relatively simple, low-motion sequences. The SoMoF Benchmark and ExPI datasets form a group of two-person scenes. These datasets have a shorter prediction horizon of approximately one second and include more active and socially complex motions, which generally result in higher forecasting errors.

Table 4 reports the percentage improvement for each model on each dataset, along with the average improvement and standard deviation within each group. A higher average value indicates better overall performance, while a lower standard deviation reflects more consistent behavior across datasets within the same group. Our proposed model achieves the highest average improvement in both categories: 54.95% for the two-person scenes and 28.56% for the three-person scenes. Furthermore, the standard deviation of its improvements is low in both groups at 1.69% and 0.1%, respectively, suggesting that the model maintains consistent performance across diverse motion scenarios.

Other models show less consistent behavior. For example, Future Motion achieves relatively strong results on the SoMoF Benchmark but performs much worse on the ExPI dataset, resulting in a high standard deviation of 14.67 percent in the two-person group. This indicates that its performance is heavily dependent on the dataset’s characteristics, limiting its generalizability. A similar pattern is observed with models such as SoMoFormer, SocialTGCN, DViTA, and TBIFormer, which exhibit noticeable variance in their performance across datasets. Even when these models do not rank the best in terms of absolute performance, their higher standard deviation values suggest limited robustness when applied to scenes with different motion dynamics or interaction complexities.

In contrast, two models that demonstrate better consistency in their generalization behavior are JRTransformer and MPFSIR. Both achieve relatively low standard deviation values across datasets in each group, indicating that their performance is more stable and less influenced by the specific characteristics of the test data. However, while they generalize more consistently, they still lag behind our proposed GCN-Transformer in terms of overall performance. Our proposed GCN-Transformer model achieves a percentage improvement over the Zero-Velocity model that is 4.7% higher than JRTransformer in the two-person group and 11.9% higher in the three-person group.

Overall, the normalized evaluation using improvements over the Zero-Velocity baseline offers a clearer and more meaningful interpretation of model performance across datasets with different characteristics. By comparing both average improvements and standard deviations, we can better understand each model’s ability to generalize beyond a single dataset, revealing that GCN-Transformer achieves the best balance of performance and consistency among all evaluated models.

## 7. Ablation Study

We conducted an ablation study on GCN-Transformer to systematically assess the impact of different components and methods on the model’s performance. This comprehensive analysis involved iteratively integrating various components and methods into the baseline model and evaluating performance at each stage. Initially, we established a baseline model comprising a Scene Module and Spatiotemporal Transformer Decoder. Subsequently, we extend the Spatiotemporal Attention Forecasting Module with Temporal-GCN, slightly enhancing model performance. Next, we introduced multi-person joint distance (MPJD) loss, further improving both short-term and long-term forecasting accuracy. Incorporating the Velocity Loss yielded a marginal improvement in overall performance, enhancing intra-sequence accuracy while slightly compromising short-term accuracy. Lastly, adding data augmentation significantly improved the model’s performance across all evaluated time intervals, representing the most substantial improvement among all modifications. Table 5 presents the evaluation results of each model on VIM and MPJPE metrics, trained exclusively on the 3DPW training set and tested on the SoMoF Benchmark validation set.

## 8. FJPTE: Final Joint Position and Trajectory Error 

**Figure 7:**
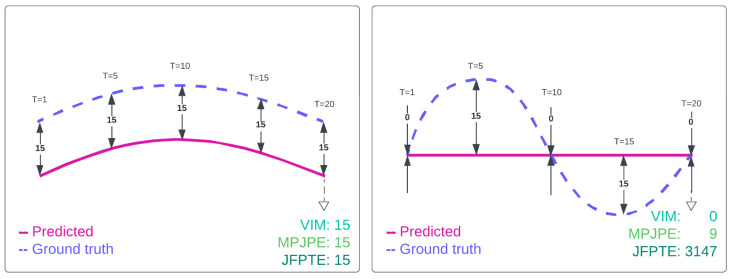
The figure illustrates an example of predicted (purple) and ground truth (blue) joint trajectories, where *T* represents the time interval, and the values between the trajectories indicate their distances at time *T*. When the trajectories are identical but have a slight offset, FJPTE yields the same results as MPJPE and VIM. However, when the trajectories diverge, the metrics produce significantly different results. MPJPE and FJPTE evaluate full joint trajectories, while VIM only evaluates the last time interval T=20.

The multitude of metrics available for pose forecasting complicates the evaluation process, as different metrics assess distinct aspects of the model’s performance. Consequently, model rankings can vary significantly depending on the chosen evaluation metric, making it challenging to identify the optimal model for the task. To address this issue, we introduce a novel metric, Final Joint Position and Trajectory Error (FJPTE), designed to consolidate the diverse objectives of pose forecasting into a single comprehensive measure. Our metric aims to capture key goals of pose forecasting, including predicting the final (N-th frame) global position (e.g., pelvis) and the trajectory of global movement leading up to that position, as well as forecasting the final pose position without global movement and its accompanying trajectory. FJPTE tackles this challenge by independently evaluating four distinct components and aggregating their results: the error in the final global position (measured by Euclidean distance), the error of the global movement trajectory (measured using the Euclidean distance of the temporal differentiation of the root joint), the error in the final pose position excluding global movement (assessed using Euclidean distance), and the trajectory error of the pose position without global movement (measured using the Euclidean distance of the temporal differentiation for all pose joints). Through this comprehensive approach, FJPTE provides a holistic assessment of a model’s performance, capturing its proficiency in capturing natural human motion dynamics and the validity of its predicted poses. An illustrative comparison of joint movement evaluation using our metric is presented in Figure 7.

**Figure 8:**
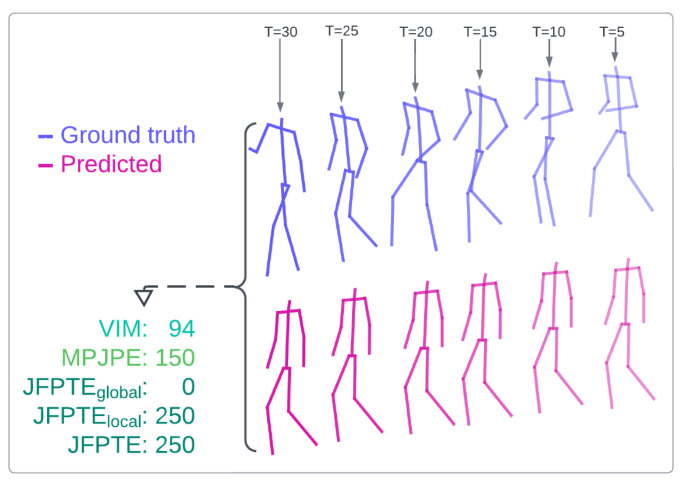
The figure illustrates an example of predicted (purple) and ground truth (blue) sequences of poses, with *T* representing the time interval. The predicted sequence demonstrates a scenario where the global position aligns well with the ground truth, but the pose remains frozen or ghost-like, floating through space, a common issue in pose forecasting. Metrics like MPJPE and VIM evaluate joint distances independently across time intervals, while the proposed FJPTE goes further by assessing joint trajectories and distinguishing between local (FJPTE_local_) and global (FJPTE_global_) movement. MPJPE and FJPTE evaluate the entire sequence, whereas VIM focuses only on the final time interval at T=30.

Additionally, Figure 8 illustrates an example where FJPTE provides a more comprehensive evaluation than MPJPE or VIM. The example shows a predicted sequence where the global position is accurate, but the pose remains frozen or ghost-like, floating unnaturally through global space, an issue that is commonly seen in pose forecasting. Unlike MPJPE, which evaluates joint distances independently across time intervals, or VIM, which focuses solely on the final interval (T=30), FJPTE comprises two key components: movement dynamics (FJPTE_local_) and global position and trajectory (FJPTE_global_). By breaking down errors into these components, FJPTE identifies whether a model struggles more with local movement dynamics or global trajectory alignment. Furthermore, by combining these errors, FJPTE enables a holistic evaluation and effective ranking of models based on their overall performance.

FJPTE is calculated as follows:

(11) $$\begin{array}{c} E_{position}\left(\hat{y},y\right)=\frac{1}{J}\sum_{j=1}^{J}||{\hat{y}\left(j\right)-y\left(j\right)||}_{2}\\ E_{trajectory}\left(\hat{Y},Y\right)=\frac{1}{T-1}\sum_{t=1}^{T-1}E_{position}\left({\hat{Y}}^{t}-{\hat{Y}}^{t+1},Y^{t}-Y^{t+1}\right)\\ E_{global}\left(\hat{Y},Y\right)=\left(E_{trajectory}\left({\hat{Y}}_{\varphi_{pelvis}},Y_{\varphi_{pelvis}}\right)+E_{position}\left({\hat{Y}}_{\varphi_{pelvis}}^{T},Y_{\varphi_{pelvis}}^{T}\right)\right)\times 1000\\ E_{local}\left(\hat{Y},Y\right)=\left(E_{trajectory}\left(\hat{Y}-{\hat{Y}}_{\varphi_{pelvis}},Y-Y_{\varphi_{pelvis}}\right)+E_{position}\left({\hat{Y}}^{T}-{\hat{Y}}_{\varphi_{pelvis}}^{T},Y^{T}-Y_{\varphi_{pelvis}}^{T}\right)\right)\times 1000\\ E_{\mathrm{FJPTE}}\left(\hat{Y},Y\right)=E_{global}\left(\hat{Y},Y\right)+E_{local}\left(\hat{Y},Y\right) \end{array}$$

where $\hat{y}$ denotes the predicted sequence, while *y* denotes the ground truth sequence. The number of joints is denoted with *J*, while the number of time intervals is denoted with *T*. ${||\cdot ||}_{2}$ denotes the Euclidean distance (L2 norm), and $\frac{1}{T-1}\sum_{t=1}^{T-1}$ represents the mean errors across all time intervals. $E_{global}\left(\hat{Y},Y\right)$ represents the global position and trajectory error between predicted and ground truth sequences measured at the pelvis joint. $E_{local}\left(\hat{Y},Y\right)$ represents the local movement dynamic errors between the predicted and ground truth sequences, excluding the pelvis joint and global movement. $E_{\mathrm{FJPTE}}\left(\hat{Y},Y\right)$ unifies local and global errors into a single metric.

We compared the models using the proposed FJPTE_local_ and FJPTE_global_ metrics on the SoMoF Benchmark test set and the reported results are shown in Table 6. The results demonstrate that GCN-Transformer significantly outperforms all other models on the FJPTE_local_ metric. This underscores GCN-Transformer’s superior ability to model human movement dynamics and interaction dynamics compared to the other models. While the overall performance hierarchy of the models remains consistent with evaluations using VIM and MPJPE metrics, LTD and JRTransformer exhibit slightly better performance in modeling movement dynamics than their immediate competitors TBIFormer and MPFSIR. When assessing the FJPTE_global_ metric, GCN-Transformer shows a slight performance gap behind SoMoFormer in long-term forecasting, indicating that SoMoFormer has a marginal edge in predicting long-term global movements. Additionally, MPFSIR emerges as a notable performer, significantly outperforming its closest competitor, Future Motion, in forecasting global positions and trajectories.

Similarly, Table 7 presents the performance of evaluated models on the ExPI test set using the proposed FJPTE_local_ and FJPTE_global_ metrics. The results indicate that GCN-Transformer consistently outperforms all other models on the FJPTE_local_ metric, except at the 120ms time interval, where JRTransformer marginally surpasses GCN-Transformer. Notably, SoMoFormer confirms that it is struggling with this dataset, while JRTransformer confirms it to be a strong contender. Another key observation is that LTD outperformed MRT on this metric compared to evaluations using the VIM and MPJPE metrics. When examining the FJPTE_global_ metric, GCN-Transformer narrowly outperforms JRTransformer, demonstrating a slight edge in overall performance despite JRTransformer’s better short-term forecasting capabilities. SoMoFormer again shows a notable decline in performance, finishing behind both JRTransformer and MPFSIR. The overall performance hierarchy of the models on the ExPI dataset remains consistent with their evaluations using the VIM and MPJPE metrics.

These results indicate that models can perform well on VIM and MPJPE metrics by focusing on global movement or movement dynamics, as models typically excel in one of these areas but not both. In contrast, FJPTE_local_ and FJPTE_global_ provide a clear distinction, making it easier to identify the best-performing models for each specific area.

Table 8 presents a comprehensive evaluation of forecasting errors using the proposed FJPTE metric, which combines FJPTE_local_ and FJPTE_global_. On the SoMoF Benchmark test set, SoMoFormer emerges as the leading model, with only GCN-Transformer*, which included the validation set during training, surpassing its performance. Most models maintain a similar performance hierarchy, as seen with VIM and MPJPE evaluations, although LTD notably outperforms both TBIFormer and MRT.

In contrast, the ExPI test set results highlight GCN-Transformer as the top performer overall. While JRTransformer slightly outperforms GCN-Transformer in short-term forecasting, GCN-Transformer consistently delivers superior results across broader time intervals. The performance ranking of other models remains largely consistent with the VIM and MPJPE evaluations. However, LTD surpasses MRT, and DViTA outperforms Future Motion, making Future Motion the lowest-performing model on the ExPI dataset using FJPTE.

To summarize, the proposed FJPTE metric significantly enhances the evaluation of pose forecasting models by providing a more detailed analysis of movement dynamics alongside global position and trajectory errors. FJPTE delivers valuable insights into how accurately predictions capture realistic motion, as demonstrated in Figure 7 and Figure 8. These examples highlight the metric’s ability to pinpoint errors in movement dynamics versus global position and trajectory deviations, offering greater clarity during evaluation. This precision is particularly impactful in applications such as surveillance, animation, and autonomous systems, where natural movement dynamics are essential for effective human–robot interaction, motion tracking, and scene understanding. By quantifying both global alignment and detailed movement nuances, FJPTE that ensures models are rewarded for producing smooth, realistic motion. Furthermore, its focus on dynamics helps mitigate common issues such as ghost-like poses or unrealistic trajectories, boosting the robustness of models in real-world, dynamic scenarios.

## 9. Limitations

While the proposed GCN-Transformer demonstrates state-of-the-art performances in multi-person pose forecasting, it is not without limitations. A key drawback of the model lies in its size; GCN-Transformer has a large number of parameters (~5.9 M), which makes it computationally expensive and memory-intensive compared to lighter models like MPFSIR (~0.15 M). While MPFSIR performs nearly as well as state-of-the-art models with significantly fewer parameters, GCN-Transformer’s parameter count is more comparable to its closest competitors, SoMoFormer (~4.9 M) and JRTransformer (~3.6 M), which mitigates this limitation to some extent.

Beyond the parameter count, the model’s computational complexity is primarily driven by the Spatiotemporal Transformer Decoder. This component scales with $O(N\cdot T^{2}\cdot d)$, where *N* is the number of individuals, *T* is the temporal sequence length, and *d* the embedding dimension. The quadratic time complexity with respect to sequence lengths is typical relative to the self-attention mechanism. The Spatial-GCN and Temporal-GCN modules are less intensive, with complexities of $O(N\cdot J^{2})$ and $O(T\cdot J^{2})$, respectively, where *J* is the number of joints.

**Figure 9:**
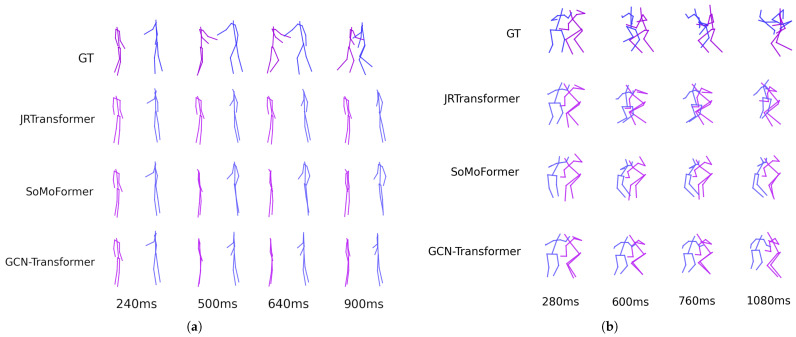
Examples from the SoMoF (**a**) and ExPI (**b**) dataset illustrating the limitations of GCN-Transformer and other models in forecasting movements not observed during training. In the SoMoF sequence (**a**), one individual approaches another, initiating a complex movement where the two prepare to spin around each other in a dance-like motion. In the ExPI sequence (**b**), two individuals perform a complex action where one lifts the other overhead to execute a backflip. Due to the absence of such intricate interactions in the training data, the models struggle to predict the dynamic sequences and instead produce a static forecast, merely repeating the last observed poses of the individuals and failing to capture the expected motion.

A more significant limitation, which is shared by GCN-Transformer and other models in the field, is the inability to forecast movements that are not represented in the training dataset. When encountering novel movements, models tend to repeat the last observed poses, resulting in frozen or static sequences. Figure 9 illustrates examples from the SoMoF and ExPI datasets, where unseen movements lead to poor forecasts. In such cases, the model fails to generalize effectively, underscoring the importance of diverse and representative training datasets to address this issue.

Another limitation of GCN-Transformer is the complexity of training due to its reliance on strong augmentations. While these augmentations improve generalization, they also necessitate longer training cycles and careful hyperparameter tuning to stabilize learning. Furthermore, despite its ability to capture interactions and dependencies between individuals, the model may struggle in scenes with highly intricate or unusual social dynamics, where interactions are more ambiguous or rare.

Lastly, the evaluation of model performance still heavily relies on benchmark datasets, which may not fully capture the diversity and variability of real-world scenarios. Consequently, there remains room for improvement in assessing and optimizing model robustness for broader applications.

These limitations provide multiple promising directions for future research. One direction is the development of more efficient, lightweight architectures that retain the ability to model complex interaction dynamics, making them suitable for deployment in real-time or resource-constrained environments. Another avenue is improving generalization relative to unseen or rare motions, which could be addressed through techniques such as data-driven motion priors, transfer learning, or motion synthesis via generative models. To support this, the field would greatly benefit from the creation of new multi-person pose forecasting datasets that include more diverse, socially rich, and dynamic interactions. Current datasets are limited in scope and variety, and expanding this benchmark space would allow models to better reflect real-world challenges and enhance their robustness in varied applications. Furthermore, improving training efficiency through adaptive enhancement strategies or self-supervised pre-training could reduce computational costs while maintaining performance.

A further limitation is that, like most multi-person forecasting models, the GCN-Transformer is trained for a fixed number of individuals per scene (e.g., two-person scenarios). When applied to datasets with a different number of individuals, minor modifications to the preprocessing pipeline are required: for example, artificially creating new sub-scenes by selecting two individuals out of a three-person scene. This design constraint is shared by all other models except SoMoFormer, which supports direct prediction for an arbitrary number of individuals without additional adjustments. Addressing this flexibility limitation without sacrificing performance in future model designs could broaden its applicability to real-world settings, where the number of individuals in a scene may vary.

## 10. Conclusions

In conclusion, this paper introduces GCN-Transformer, a novel model for multi-person pose forecasting that leverages the synergies of Graph Convolutional Network and Transformer architectures. We conducted a thorough evaluation of GCN-Transformer alongside other state-of-the-art models, presenting results on the CMU-Mocap, MuPoTS-3D, SoMoF Benchmark, and ExPI datasets using the VIM and MPJPE metrics. The results on the CMU-Mocap and MuPoTS-3D datasets, which feature three-person interaction scenes with generally simpler and lower interaction motions compared to ExPI, show that our model consistently achieves state-of-the-art performance across both datasets, demonstrating its robustness across varying levels of interaction complexities and different numbers of people in the scene. The results on the SoMoF Benchmark should be cautiously interpreted due to the dataset’s inherent randomness, attributed to the sequences recorded with a moving camera. This introduces complexities as models must predict human and camera movements, often perceived as erratic. To mitigate this, we additionally evaluated all models on the ExPI dataset, featuring challenging actions performed by two couples without camera movement. Conclusively, GCN-Transformer consistently outperforms existing state-of-the-art models on all datasets.

Furthermore, we propose a novel evaluation metric, FJPTE, which comprehensively assesses pose forecasting errors by accounting for both local movement dynamics (FJPTE_local_) and global movement (FJPTE_global_). These components are computed based on errors at the final position and along the trajectory leading up to that point. Our evaluation of all models using FJPTE reveals that GCN-Transformer excels in capturing both intricate movement dynamics and accurate global position trajectory, where it consistently achieves state-of-the-art results.

The superior performance of GCN-Transformer can be attributed to its hybrid architecture that allows the model to capture fine-grained spatial dependencies within individuals while also modeling long-range temporal and social interactions across people in the scene. The attention mechanism further enhances robustness by enabling the model to focus dynamically on relevant joints and individuals, which is particularly effective in handling socially complex behaviors, such as those found in the ExPI dataset. As a result, GCN-Transformer demonstrates strong generalization across varying motion types and interaction intensities, outperforming prior approaches that lack either spatial specificity or long-term temporal modeling capacity.

Overall, the success of the proposed GCN-Transformer underscores its potential to drive the field of multi-person pose forecasting, with promising applications in human–computer interaction, sports analysis, and augmented reality. Beyond its empirical performance, this work introduces a modular modeling and evaluation perspective for interaction-rich forecasting, where generating socially coherent pose sequences and evaluating them using trajectory and position-aware metrics are addressed together. These design choices contribute toward advancing more expressive, generalizable, and testable architectures for multi-person pose forecasting. As future work, we aim to explore further enhancements for GCN-Transformer’s architecture, including the integration of activity recognition to aid in pose forecasting, and we will investigate its applicability to real-world scenarios.

## Figures and Tables

**Table 1:** Performance comparison on the test sets of the CMU-Mocap and MuPoTS-3D datasets, featuring three-person scenes. Results are reported using the MPJPE metric (in meters), where lower values indicate better joint position prediction accuracy. Our proposed GCN-Transformer consistently achieves state-of-the-art results, outperforming all competing models on both datasets.

Method	MPJPE Metric								
	CMU-Mocap Test Set				MuPoTS-3D Test Set				Average Overall
	1 s	2 s	3 s	Overall	1 s	2 s	3 s	Overall	
Zero Velocity	5.55	9.23	12.30	9.03	2.05	3.43	4.57	3.35	6.29
MRT [15]	4.46	7.94	10.94	7.78	1.87	3.40	5.04	3.44	5.61
SoMoFormer [16]	4.50	8.15	11.27	7.79	1.69	3.02	4.15	2.95	5.37
Future Motion [13]	4.08	7.24	10.21	7.18	1.98	3.40	4.57	3.31	5.25
JRTransformer [18]	4.08	7.47	10.47	7.34	1.61	2.90	4.06	2.86	5.16
LTD [3]	4.03	7.06	9.91	7.00	1.75	2.98	4.10	2.94	4.97
MPFSIR [17]	3.94	7.04	9.87	6.95	1.67	2.87	3.93	2.82	4.89
**GCN-Transformer (our)**	**3.53**	**6.58**	**9.25**	**6.46**	**1.39**	**2.41**	**3.39**	**2.40**	**4.43**

Best results in each column are highlighted in bold.

**Table 2:** Performance comparison on the SoMoF Benchmark test set featuring two-person scenes, using the VIM and MPJPE metrics, where lower values indicate better performances. Our proposed model, GCN-Transformer, achieves state-of-the-art results. The model marked with an asterisk (*) incorporated the validation dataset during training and currently leads the official SoMoF Benchmark leaderboard at https://somof.stanford.edu.

Method	Metrics											
	VIM						MPJPE					
	100 ms	240 ms	500 ms	640 ms	900 ms	Overall	100 ms	240 ms	500 ms	640 ms	900 ms	Overall
Zero Velocity	29.35	53.56	94.52	112.68	143.10	86.65	55.28	87.98	146.10	173.30	223.16	137.16
DViTA [12]	17.40	35.62	72.06	90.87	127.27	68.65	32.09	54.48	100.03	124.07	173.01	96.74
LTD [3]	18.07	34.88	68.16	85.07	116.83	64.60	33.57	55.21	97.57	119.58	163.69	93.92
TBIFormer [19]	17.62	34.67	67.50	84.01	116.38	64.03	32.26	53.65	95.61	117.22	160.99	91.94
MRT [15]	15.31	31.23	63.16	79.61	111.86	60.24	27.97	47.64	87.87	108.93	151.96	84.88
SocialTGCN [20]	12.84	27.41	58.12	74.59	107.19	56.03	23.10	40.24	76.91	96.89	139.01	75.23
JRTransformer [18]	11.17	25.73	56.50	73.19	106.87	54.69	18.44	35.38	72.26	92.42	135.12	70.73
MPFSIR [17]	11.57	25.37	54.04	69.65	101.13	52.35	20.31	35.69	69.58	88.36	128.37	68.46
Future Motion [13]	10.76	24.52	54.14	69.58	100.81	51.96	18.66	34.38	69.76	88.91	129.18	68.18
SoMoFormer [16]	10.45	23.10	49.76	64.30	**93.34**	48.19	17.63	32.42	63.86	81.20	**117.97**	62.62
**GCN-Transformer (our)**	**10.14**	**22.54**	**48.81**	**63.67**	94.94	**48.02**	**17.11**	**31.48**	**62.62**	**80.14**	118.14	**61.90**
GCN-Transformer * (our)	**9.82**	**21.80**	**46.61**	**60.88**	**91.95**	**46.21**	**16.41**	**30.36**	**60.31**	**76.94**	**113.36**	**59.48**

Best results in each column are highlighted in bold.

**Table 3:** Performance comparison on the ExPI test set featuring two-person scenes using the VIM and MPJPE metrics, where lower values indicate better performance. Our proposed model, GCN-Transformer, achieves state-of-the-art results on both metrics.

Method	Metrics											
	VIM						MPJPE					
	120 ms	280 ms	600 ms	760 ms	1080 ms	Overall	120 ms	280 ms	600 ms	760 ms	1080 ms	Overall
Zero Velocity	25.61	48.66	84.39	97.41	118.10	74.84	46.16	74.66	124.32	145.22	181.33	114.34
DViTA [12]	15.44	35.27	74.43	91.44	119.51	67.22	28.31	51.63	100.85	124.49	167.98	94.65
LTD [3]	16.22	32.94	62.73	74.60	92.84	55.87	28.83	48.73	87.37	104.82	135.61	81.07
TBIFormer [19]	16.96	35.09	67.95	81.22	103.02	60.85	30.59	52.55	95.63	115.19	150.33	88.86
MRT [15]	15.32	32.07	61.84	74.04	94.59	55.57	27.79	47.91	87.01	104.80	137.22	80.95
SocialTGCN [20]	16.79	32.71	62.61	75.24	99.15	57.30	31.14	50.58	89.18	106.95	140.68	83.71
JRTransformer [18]	8.40	21.14	46.20	57.63	76.94	42.06	13.57	28.01	58.47	73.27	101.04	54.87
MPFSIR [17]	9.15	23.05	52.31	65.49	92.46	48.49	15.56	30.55	64.84	81.81	114.94	61.54
Future Motion [13]	16.94	34.83	68.45	83.33	108.03	62.32	30.51	52.37	96.06	116.88	156.04	90.37
SoMoFormer [16]	9.43	23.88	54.78	68.71	92.38	49.84	15.22	31.08	67.33	85.37	119.37	63.67
**GCN-Transformer (our)**	**8.32**	**20.84**	**44.56**	**54.81**	**74.66**	**40.64**	**13.37**	**27.63**	**57.27**	**71.25**	**97.71**	**53.45**

Best results in each column are highlighted in bold.

**Table 4:** Percentage improvement over the Zero-Velocity baseline across all evaluated datasets, grouped by 3-person and 2-person scenes. Each value indicates the relative reduction in MPJPE, where higher values represent better performance. The table includes average improvements (Avg) and the standard deviation (Std) to reflect generalization consistency across datasets within each group. The best values in each group are shown in bold. The percentage improvement is computed as follows: $\mathrm{Improvement}=\left(\mathrm{Zero}\;\mathrm{Velocity}-\mathrm{Method}\right)/\mathrm{Zero}\;\mathrm{Velocity}\times 100\%$.

Method	Percentage Improvements over Zero-Velocity Baseline (Based on Overall MPJPE Across All Datasets)							
	2-Person Scenes				3-Person Scenes			
	SoMoF ↑	ExPI ↑	Avg (%) ↑	Std (%) ↓	CMU-Mocap ↑	MuPoTS-3D ↑	Avg (%) ↑	Std (%) ↓
Zero Velocity	0.0	0.0	0.0	0.0	0.0	0.0	0.0	0.0
DViTA [12]	29.47	17.22	23.34	6.12				
TBIFormer [19]	32.97	22.29	27.63	5.34				
LTD [3]	31.52	29.10	30.31	**1.21**	22.48	12.24	17.36	5.12
MRT [15]	38.12	29.21	33.66	4.45	13.84	-2.69	5.58	8.26
Future Motion [13]	50.30	20.96	35.63	14.67	20.49	1.19	10.84	9.65
SocialTGCN [20]	45.15	26.79	35.97	9.18				
MPFSIR [17]	50.09	46.18	48.14	1.96	23.03	15.82	19.42	3.61
SoMoFormer [16]	54.35	44.31	49.33	5.02	13.73	11.94	12.84	0.90
JRTransformer [18]	48.44	52.01	50.22	1.78	18.72	14.63	16.68	2.04
**GCN-Transformer (our)**	**56.64**	**53.26**	**54.95**	1.69	**28.46**	**28.66**	**28.56**	**0.1**

Best results in each column are highlighted in bold. Arrows next to the column names indicate the direction of better performance: ↑ means higher is better, ↓ means lower is better.

**Table 5:** The ablation study results are derived from the SoMoF Benchmark validation set and presented in VIM (top) and MPJPE (bottom) metrics. The baseline model comprises Scene Module and the Spatiotemporal Transformer Decoder, with subsequent additions incrementally incorporated into the model. All models are trained solely on the SoMoF Benchmark training dataset, excluding AMASS.

Metric	Method	100 ms	240 ms	500 ms	640 ms	900 ms	Overall
VIM	Baseline	15.39	28.53	55.90	68.72	93.92	52.49
	+ Temporal-GCN	12.69	28.96	58.96	69.74	89.56	51.98
	+ MPJD loss	11.08	28.80	57.52	67.55	87.95	50.58
	+ Velocity loss	12.21	28.30	56.12	66.42	87.67	50.14
	+ Augmentation	**7.56**	**19.66**	**44.72**	**56.08**	**75.12**	**40.63**
MPJPE	Baseline	31.81	45.19	77.03	93.68	127.60	75.06
	+ Temporal-GCN	23.99	41.47	79.33	96.38	127.61	73.76
	+ MPJD loss	18.09	37.54	76.08	92.69	123.51	69.58
	+ Velocity loss	22.79	39.90	75.28	91.15	121.77	70.18
	+ Augmentation	**11.68**	**24.35**	**53.50**	**68.34**	**96.97**	**50.97**

Best results in each column are highlighted in bold.

**Table 6:** Comparison of performance on the SoMoF Benchmark test set using the proposed FJPTE metric, with lower values indicating superior performance. The table distinguishes between FJPTE_local_ and FJPTE_global_ errors, with FJPTE_local_ representing movement dynamics errors and FJPTE_global_ measuring global position and trajectory errors. The asterisk (*) denotes the model that integrated the validation dataset during training.

Method	Components of Proposed FJPTE Metric											
	Proposed FJPTE_local_						Proposed FJPTE_global_					
	100 ms	240 ms	500 ms	640 ms	900 ms	Overall	100 ms	240 ms	500 ms	640 ms	900 ms	Overall
Zero Velocity	65.36	97.18	142.35	158.79	178.72	128.48	91.12	146.51	241.69	284.08	363.52	225.38
DViTA [12]	55.15	91.84	147.91	168.07	194.29	131.45	47.60	81.35	162.46	212.71	319.11	164.65
LTD [3]	48.96	78.96	127.59	145.98	170.41	114.38	52.86	88.66	159.64	201.40	290.96	158.70
TBIFormer [19]	55.24	88.28	138.76	156.81	178.97	123.61	51.19	84.53	150.47	190.78	283.36	152.07
MRT [15]	56.38	90.59	143.17	162.19	186.11	127.69	46.74	77.70	147.95	189.65	279.84	148.37
SocialTGCN [20]	51.50	83.54	137.45	157.54	183.19	122.64	39.76	65.92	132.28	175.90	271.09	136.99
JRTransformer [18]	41.20	72.47	124.75	145.87	174.81	111.82	26.87	54.81	122.92	166.64	264.94	127.24
MPFSIR [17]	43.53	75.36	127.59	148.60	180.67	115.15	27.37	51.27	109.84	151.17	248.05	117.54
Future Motion [13]	42.74	72.22	122.18	140.77	165.83	108.75	31.04	54.72	117.86	158.93	249.45	122.40
SoMoFormer [16]	37.69	65.48	111.48	128.79	154.44	99.58	26.13	48.37	**104.01**	**139.66**	**217.92**	**107.22**
**GCN-Transformer (our)**	**37.22**	**63.78**	**109.06**	**126.12**	**152.72**	**97.78**	**24.35**	**47.42**	107.12	146.38	234.51	111.96
GCN-Transformer * (our)	**36.76**	**62.29**	**104.96**	**121.68**	**147.97**	**94.73**	**23.63**	**45.89**	**102.05**	**138.45**	228.94	107.79

Best results in each column are highlighted in bold.

**Table 7:** Comparison of performances on the ExPI test set using the proposed FJPTE metric, with lower values indicating superior performance. The table distinguishes between FJPTE_local_ and FJPTE_global_ errors, with FJPTE_local_ representing movement dynamics errors and FJPTE_global_ measuring global position and trajectory errors.

Method	Components of Proposed FJPTE Metric											
	**Proposed FJPTE_local_**						**Proposed FJPTE_global_**					
	120 ms	280 ms	600 ms	760 ms	1080 ms	Overall	120 ms	280 ms	600 ms	760 ms	1080 ms	Overall
Zero Velocity	76.63	119.52	182.09	205.19	240.31	164.75	79.80	127.56	201.88	230.77	280.05	184.01
DViTA [12]	56.91	101.25	176.21	206.20	252.27	158.57	45.58	83.58	164.19	202.36	271.01	153.34
LTD [3]	60.27	97.73	159.16	182.82	217.66	143.53	47.42	80.89	141.84	169.41	215.70	131.05
TBIFormer [19]	67.38	109.04	174.85	200.29	239.29	158.17	50.23	86.97	155.57	184.96	238.15	143.18
MRT [15]	65.77	107.77	173.87	199.12	236.71	156.65	43.80	75.45	133.75	162.58	214.24	125.96
SocialTGCN [20]	72.62	110.05	174.62	201.84	247.24	161.27	52.04	83.27	149.11	178.12	237.98	140.10
JRTransformer [18]	**37.98**	71.62	130.94	155.35	197.44	118.67	**26.21**	**52.63**	102.44	126.11	168.75	95.23
MPFSIR [17]	41.12	77.88	145.78	174.01	225.03	132.76	27.21	54.68	112.28	140.63	207.33	108.43
Future Motion [13]	64.87	105.26	175.12	206.69	247.48	159.88	48.70	86.51	160.21	197.70	270.41	152.71
SoMoFormer [16]	41.91	80.52	150.92	179.58	224.17	135.42	28.82	57.92	118.39	148.45	204.18	111.55
**GCN-Transformer (our)**	38.39	**71.60**	**125.41**	**146.24**	**181.17**	**112.56**	26.67	52.74	**100.23**	**122.83**	**172.73**	**95.04**

Best results in each column are highlighted in bold.

**Table 8:** Comparison of performance on the SoMoF Benchmark test set (left) and the ExPI test set (right) using the proposed FJPTE metric, where lower values indicate better performance. The table presents FJPTE metric, combining FJPTE_local_ and FJPTE_global_ errors for a comprehensive performance evaluation. Our model achieves state-of-the-art results on the FJPTE metric. The asterisk (*) indicates models that integrated the validation dataset during training.

Method	Proposed FJPTE Metric											
	SoMoF Benchmark						ExPI					
	100 ms	240 ms	500 ms	640 ms	900 ms	Overall	120 ms	280 ms	600 ms	760 ms	1080 ms	Overall
Zero Velocity	156.48	243.69	384.04	442.87	542.24	353.86	156.43	247.07	383.97	435.95	520.36	348.76
DViTA [12]	102.75	173.20	310.36	380.78	513.40	296.10	102.48	184.82	340.40	408.56	523.29	311.91
LTD [3]	101.82	167.62	287.23	347.38	461.37	273.08	107.69	178.62	301.01	352.23	433.36	274.58
TBIFormer [19]	106.43	172.81	289.23	347.59	462.33	275.68	117.61	196.01	330.42	385.25	477.45	301.35
MRT [15]	103.11	168.29	291.12	351.84	465.95	276.06	109.58	183.22	307.63	361.70	450.95	282.62
SocialTGCN [20]	91.26	149.46	269.73	333.44	454.28	259.63	124.66	193.32	323.73	379.95	485.22	301.38
JRTransformer [18]	68.07	127.29	247.68	312.51	439.75	239.06	**64.19**	**124.25**	233.39	281.46	366.19	213.90
MPFSIR [17]	70.91	126.63	237.44	299.78	428.72	232.69	68.33	132.56	258.06	314.65	432.35	241.19
Future Motion [13]	73.78	126.94	240.04	299.70	415.28	231.15	113.57	191.77	335.33	404.39	517.89	312.59
SoMoFormer [16]	63.82	113.85	**215.50**	**268.45**	**372.35**	**206.79**	70.73	138.44	269.31	328.03	428.35	246.97
**GCN-Transformer (our)**	**61.57**	**111.21**	216.17	272.50	387.22	209.73	65.07	124.34	**225.64**	**269.07**	**353.90**	**207.60**
GCN-Transformer * (our)	**60.39**	**108.19**	**207.01**	**260.13**	**376.91**	**202.53**	-	-	-	-	-	-

Best results in each column are highlighted in bold.

## Data Availability

The original data presented in the study are openly available at https://github.com/RomeoSajina/GCN-Transformer (accessed on 2 February 2025).
